# Achieving Reliability in Cloud Computing by a Novel Hybrid Approach

**DOI:** 10.3390/s23041965

**Published:** 2023-02-09

**Authors:** Muhammad Asim Shahid, Muhammad Mansoor Alam, Mazliham Mohd Su’ud

**Affiliations:** 1 Malaysian Institute of Information Technology, Universiti Kuala Lumpur, Kuala Lumpur 50250, Malaysia; 2 Faculty of Computing, Riphah International University, Sector I-14, Hajj Complex, Islamabad 46000, Pakistan; 3 School of Computer Science, University of Technology Sydney, 15 Broadway, Ultimo, NSW 2007, Australia; 4 Persiaran Multimedia, Multimedia University, Cyberjaya 63100, Malaysia

**Keywords:** cloud computing, delta-checkpointing, fault tolerance, fault classification and prediction, machine learning, reliability, Weibull distribution

## Abstract

Cloud computing (CC) benefits and opportunities are among the fastest growing technologies in the computer industry. Cloud computing’s challenges include resource allocation, security, quality of service, availability, privacy, data management, performance compatibility, and fault tolerance. Fault tolerance (FT) refers to a system’s ability to continue performing its intended task in the presence of defects. Fault-tolerance challenges include heterogeneity and a lack of standards, the need for automation, cloud downtime reliability, consideration for recovery point objects, recovery time objects, and cloud workload. The proposed research includes machine learning (ML) algorithms such as naïve Bayes (NB), library support vector machine (LibSVM), multinomial logistic regression (MLR), sequential minimal optimization (SMO), K-nearest neighbor (KNN), and random forest (RF) as well as a fault-tolerance method known as delta-checkpointing to achieve higher accuracy, lesser fault prediction error, and reliability. Furthermore, the secondary data were collected from the homonymous, experimental high-performance computing (HPC) system at the Swiss Federal Institute of Technology (ETH), Zurich, and the primary data were generated using virtual machines (VMs) to select the best machine learning classifier. In this article, the secondary and primary data were divided into two split ratios of 80/20 and 70/30, respectively, and cross-validation (5-fold) was used to identify more accuracy and less prediction of faults in terms of true, false, repair, and failure of virtual machines. Secondary data results show that naïve Bayes performed exceptionally well on CPU-Mem mono and multi blocks, and sequential minimal optimization performed very well on HDD mono and multi blocks in terms of accuracy and fault prediction. In the case of greater accuracy and less fault prediction, primary data results revealed that random forest performed very well in terms of accuracy and fault prediction but not with good time complexity. Sequential minimal optimization has good time complexity with minor differences in random forest accuracy and fault prediction. We decided to modify sequential minimal optimization. Finally, the modified sequential minimal optimization (MSMO) algorithm with the fault-tolerance delta-checkpointing (D-CP) method is proposed to improve accuracy, fault prediction error, and reliability in cloud computing.

## 1. Introduction

Motivation. CC debuted in information technology and has since evolved into a popular business model for providing IT infrastructure, components, and applications [1]. The five distinct characteristics of CC are on-demand self-service, extensive network access, resource pooling, rapid elasticity, and measured service. In addition, four deployment models are available: private clouds, community clouds, public clouds, and hybrid clouds. In addition, it provides three service models: SaaS, PaaS, and IaaS [2].

In providing cloud security assistance, FT is a significant challenge. Several previous studies attempted to incorporate the various FT frameworks and cloud solutions proposed, but certain accounts proved to be limited. The ability of a system to perform properly in the presence of internal faults is referred to as FT [3,4]. In fault tolerance, there are four types of system faults: transient faults, intermittent faults, permanent faults, and Byzantine faults [5,6,7]. The FT technique taxonomy is divided into three major groups: reactive methods (RAMs), proactive methods (PRMs), and resilient methods (RMs) [5]. Methods in the above taxonomy include checkpointing (CP)/restarting, replication, retry, task resubmission, custom exception handling, rescue workflow, N-version and recovery block, software rejuvenation, preemptive migration, self-healing, prediction, ML, and fault induction [3].

Reliability has always been a major challenge in distributed systems. It is critical to provide highly accessible and dependable CC services to maintain client confidence and satisfaction while avoiding revenue losses. Although various solutions for cloud reliability have been proposed, there are no comprehensive studies that cover all aspects of the problem [8]. Improving cloud service reliability is a critical feature of CC that has received a great deal of attention from the research community [9].

The Antarex secondary dataset is made up of trace data collected from the homonymous experimental HPC system at ETH Zurich during fault injection to perform ML-based fault detection experiments on HPC systems. The dataset is divided into two sections: one for CPU and memory-related benchmark apps and fault programs and another for hard-drive-related apps and fault programs. The Antarex dataset is divided into four folders: one for each dataset block, namely CPU/memory and HDD, in single-core and multi-core forms [10]. The Weibull distribution approach is used to generate the primary dataset. The Weibull distribution is also commonly used as a time-to-failure model in reliability. It extends the exponential model by incorporating non-constant failure rate functions. This includes both rising and decreasing failure rate functions and has been used effectively to explain both initial burning failures and wear-out failures [11].

ML has played an active part in the RAMs area, mapping the recovery time to a function that can be improved (i.e., by converging the recovery time to a fraction of milliseconds). The recovery time will decrease as the system learns to deal with new errors. Researchers have recently become more interested in resilient approaches. The resilience of a system is defined as the speed with which it can recover and resume regular operation following a system outage or failure. RSMs include techniques for responding to clients despite failure, monitoring system state, and learning and adapting from faults and predictions. The learning and adaptation of a system are based on ML in RSMs. Resilient approaches include techniques that deal with the ability to respond to clients despite failure, monitoring system status, learning and adapting from faults, and forecasting. In resilient approaches, the learning and adaptability of a system based on ML are used [5].

Contributions. Our work makes numerous contributions. We began by acquiring the HPC fault dataset and evaluating a fault classification method based on supervised ML. This dataset and all test environment details are publicly available for use by the community. The Antarex secondary dataset is based on trace data from the homonymous experimental HPC system at ETH Zurich during fault injection, which was used to undertake ML-based fault prediction studies for researchers. The dataset was then separated into two sections: one for CPU and memory-related benchmark apps and fault programs and another for hard-drive-related applications and fault programs. The Antarex dataset has four folders: one for each dataset block, namely CPU/M\memory and HDD, in single-core and multi-core forms [10].

Second, we generated a primary dataset through the Weibull distribution approach. The Weibull distribution is also often employed as a time-to-failure model for reliability. It extends the exponential model by including non-constant failure rate functions. This contains both rising and falling failure rate curves and has been successfully utilized to explain both initial burnings and wear-out failures [11]. We coded different parameters in the Java platform for primary data generated using the Weibull distribution approach.

Third, our evaluation is based on the Antarex secondary datasets that we acquired from the ZONODO website and generated primary datasets using the Weibull distribution approach [10,11]. We present the results of our experiments, whose purpose was for fault classification and fault prediction to detect which of the ML algorithms gives better results in terms of high accuracy and less fault prediction.

As a fourth and final contribution, reliability was achieved using the MSMO classifier and D-CP fault-tolerance approach to achieve high accuracy, less fault prediction, and successful execution of VMs that have a good impact on users related to the CC environment.

Organization. The remainder of the paper is structured as follows. The first section provides an overview of this research study. In the following section, we contextualize our work on literature review, and in Section 3, we demonstrate the problem statement. Section 4 discusses the research methodology, Section 5 discusses results and findings, Section 6 is based on discussion, and Section 7 concludes this research.

## 2. Literature Review

Shahid et al. [12] suggested that in recent years, CC has emerged as a distinct trend. Distributed systems have evolved into large-scale computer networks as a result. Cloud computing companies such as International Business Management Corporation (IBM), Amazon, Yahoo, and Google provide cloud services to customers worldwide. End users are not required to install programs on their local personal computers (PCs) under this novel paradigm; instead, apps and services are delivered to them on demand. CC is facing several challenges. Data protection, data recovery and availability, administrative capabilities, regulatory compliance, security, and the ability to adjust the burden, control execution, fault tolerance, cloud computing governance, interoperability, and portability are examples.

Gupta et al. [13] suggested that FT is a difficult research area that is being pursued alongside distributed grid computing. This is due to the long time it takes computer-intensive grid systems to solve a single problem. The FT of CC is flexible, which leads to unanticipated tasks, which leads to faults. To improve CC dependability and resilience, defects should be properly investigated and managed. In computer systems, there are two types of FT: hardware and software.

Shahid et al. [3] suggested that the taxonomy types of FT techniques be divided into three key groups: RAMs, PRMs, and RSMs. Cloud FT advice in the future will emphasize clever and resilient solutions. Previously, many FT researchers used ML to demonstrate intelligence and resilience in various ways. As a third group, they added resilient approaches on their own.

Mukwevho and Celik [14,15] suggested that downtime in the cloud might be defined as not dropping the in-storage conference state in the case of a disaster, such as a host server breakdown or a network system disaster, rather than stopping the service entirely. A detailed outage at one data center may have an impact on other businesses. Each organization has different SLAs for clouds, and the FT supplier must guarantee that the SLAs are satisfied for all organizations.

Edemo [16] recommended this strategy as an effective reactive method, particularly for long-running programs. CP is the practice of maintaining app status after any effective completion or storing the preliminary failure-free state. This strategy is implemented at several levels, including the device, app, and library levels.

Kamiri and Mariga [17] suggested that ML is a subfield of artificial intelligence that deals with the creation of algorithms and procedures that allow a computer to learn and gain intelligence through experience. The research methodology used in ML research is critical because it influences the accuracy and dependability of the results. ML models learn from historical data, which can be primary or secondary in nature. As a result, there is a vast knowledge base from which robots can learn and make decisions.

Sarker [18] suggested that the process of learning a function that translates input to output was introduced using sample input–output pairs. It uses labeled training data and a set of training examples to infer a function. When specific goals are specified to be achieved from a specific set of inputs, i.e., a task-driven method, supervised learning occurs. The most common supervised tasks are classification (data separation) and regression (fitting data). For example, supervised learning is used to predict the class label or sentiment of a piece of text, such as a tweet or product review.

Butt et al. [19] investigated ML as the logical evaluation of computations and quantifiable models used by computer systems to perform a particular attempt without the need for explicit instructions based on models and acceptance. It falls under the umbrella of computerized reasoning. ML is so important in the cloud that it will be used by all clouds soon.

Sun et al. [20] suggested that ML has recently grown at a breakneck pace, attracting a large number of academics and practitioners. It has emerged as one of the most prominent research areas, with applications in a wide range of industries including machine translation, speech recognition, image recognition, recommendation systems, and so on.

Kochhar et al. [21] suggested the NB classifier is one of the most useful ML algorithms. The NB classifier is based on the Bayes theorem, which requires significant independence (naïve) between qualities or features (predictors). Because it requires little work to develop and has no complicated repeating parameter setting or computation, the naïve Bayesian classification model is very useful for very large datasets. Despite its simplicity, the NB classifier is one of the most widely used algorithms because it frequently outperforms more complicated and refined classification algorithms.

Chang and Lin [22] proposed that LIBSVM is a support vector machines library. The goal is to make applying SVM to applications as simple as possible for users. LIBSVM has been widely used in ML and other fields. LIBSVM has grown to be one of the most widely used SVM programs. LIBSVM provides support for a variety of SVM formulations for classification, regression, and distribution estimation. LIBSVM is widely used in numerous fields.

Mohamad [23] found that based on many independent factors, MLR is used to estimate the probability of multiple possible outcomes for a categorical dependent variable with more than two categories. The MLR model compares various categories using a combination of binary logit models. The multinomial logit model is composed of k-1 binary logit models that assess the influence of predictors on the likelihood of success in that category for k response variable categories.

C.R. LI and J. GUO [24] proposed that the SMO limits B to only two multipliers that can be calculated analytically and do not require any extra matrix storage. There are two methods for determining which multipliers to optimize. The first heuristic prioritizes unbound multipliers that are more likely to violate the KKT specifications. The second-choice heuristic, after selecting the first Lagrange multiplier, selects the second Lagrange multiplier that maximizes the difference between the two prediction errors. To save training time, the SMO technique is based on a single-program multiple data (SPMD) paradigm. It divides the entire dataset into smaller subsets and uses several processors to update the error array of each subset in parallel.

Sen et al. [25] suggested that the KNN saves all available records and predicts the class of new occurrences in probability using similarity measures from the nearest neighbors. Unlike other classification techniques that construct a mapping function or internal model, this classification technique is known as a lazy learning method because it stores the data members in inefficient data structures such as hash tables, reducing the computation cost to check and apply the appropriate distance function between the new observation and all k amount of different data points stored and then come to any conclusion about the label of the new data point. The results are generated by applying simple majority support to the KNN of each new data point.

In the work by Attallah et al. [26], the proposed methodology tolerates VM CPU faults to achieve maximum CC infrastructure reliability and availability. CPU faults can occur during VM operation. The proposed model’s main goal is to track changes in CPU utilization and make a decision when a high value of CPU utilization is detected. It either moves the faulty VM to a different destination host or manages loads on the destination host so that the faulty VM can be moved.

S. Suguna and K. Devi [27] suggested virtual machine fault tolerance (VMFT). The machine tolerates failure in this method based on the VMs reliability. It delivers reliability and availability while also shortening service times. When the application is calculated on a VM, the VM that produces the proper logical output in the shortest amount of time is regarded as the best VM among all VMs, and that VM is used for further application processing. The suggested VMFT approach is implemented using a cloud sim tool. The time it takes to execute the program is used to measure the reliability of a single-node VM. The node that returns the result on time is designated as the reliable VM.

Sarker [18] the proposed RF classifier is a well-known ensemble classification approach used in machine learning and data science in a wide range of application fields. This method employs a parallel ensemble, which entails fitting multiple decision tree classifiers to different data sets sub-samples concurrently, with the conclusion or final result determined by majority voting or averages. As a result, over-fitting is reduced, and forecast accuracy and control are improved. As a result, the RF learning model with multiple decision trees outperforms a single decision tree model regularly. It generates a series of decision trees with a controlled variance using a combination of bootstrap aggregation (bagging) and random feature selection. Table 1 shows a summary of the literature review.

## 3. Problem Statement

Reliability is a continuous metric that changes with each computing step. One of the most important service characteristics is reliability, which must be met in CC for a stable operation. The main backup duplication is a critical FT software strategy used to meet reliability requirements. The dependability of overall task completion is the result of specific activities, and for too many thousands or millions of computing operations, this can quickly become a fading variety. A cloud system’s reliability is an assessment of how effectively the cloud system provides the service to the user based on the criteria listed above [8].

There is a need to design and implement ML models that can resolve FT issues by acquiring high accuracy, less fault prediction, and achieving optimum reliability based on successfully running VMs without any failure.

### Mathematical Equation for Reliability

In complex systems, the analyst requires a mathematical approach to determine the importance of each VM. Reliability is an appropriate measure for determining the relative importance of each virtual machine to the overall system reliability. The reliability importance, IRi, of component i in an n-VM system is given as follows [8]:

(1) $$RIi\left(t\right)=\;\frac{\partial Rs\left(t\right)}{\partial Ri\left(t\right)}$$

where Rs (t) is the reliability at a given time, t; and Ri (t) is the VM reliability at the same time, t [8].

RI measures the rate of change of system reliability in relation to VM reliability at a given time t. The RI can also be used to calculate the likelihood that a component will cause a system failure at time t. The calculated reliability importance in Equation (1) can be influenced by both reliability and the current position of a system component [8].

## 4. Research Methodology

This section focuses on the proposed methodology. In this section, the research design, data collection procedure, and data analysis techniques are all explained in detail. The architecture of the data analysis techniques is also incorporated and explained.

### 4.1. Research Design

The following research design was followed.

#### 4.1.1. Data Collected and Generated

The secondary dataset contains trace data collected from the ETH Zurich homonymous, experimental HPC system, and the generated primary dataset contains repair and failure virtual machine data to conduct an ML-based approach for FT reliability in CC.

#### 4.1.2. Machine Learning Algorithms

This research is based on supervised ML algorithms to achieve high accuracy and less fault prediction error. It is defined by its use of labeled datasets to train algorithms to classify data or predict outcomes accurately.

#### 4.1.3. Fault-Tolerance Approach

In this research, FT is used to identify the failure and repair of VMs. Virtual machines are used in a CC system to handle user requests for services. A user request cannot be completed if the virtual machine fails. D-CP mechanisms are used to mitigate the impact of VM failure.

#### 4.1.4. Reliability

In CC, reliability is defined as a cloud computing system’s capacity to complete the intended job or deliver a necessary service for a certain amount of time under predetermined conditions. In this research, reliability is achieved based on ML and the FT method. Reliability means that all VMs have been run successfully without any failure.

### 4.2. Implementation View of Research Framework

The research framework diagram was designed to understand the flow of the proposed research. In the beginning, the secondary data acquisition was completed from external sources, and primary data were generated through the Weibull distribution approach. The secondary data set was cleaned out and then processed. The primary dataset was clean. 

**Figure 1:**
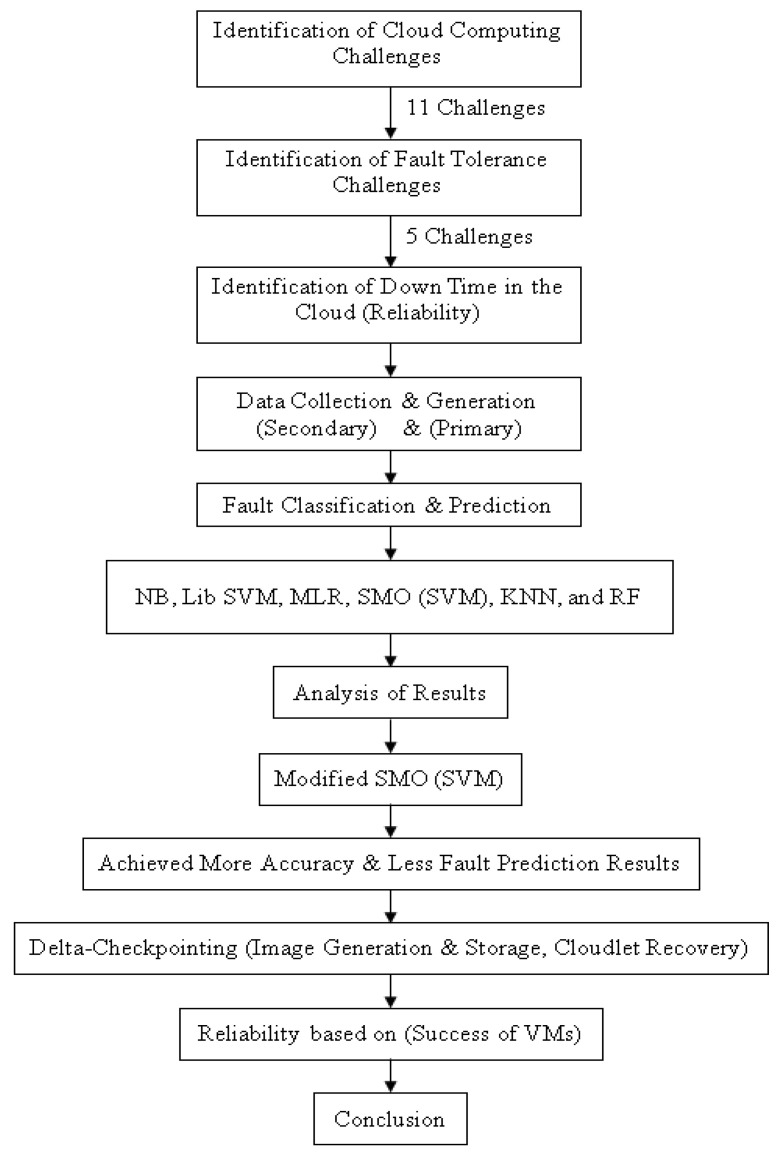
Implementation View of Research Framework.

Figure 1 demonstrates that a genuine, competent, and effective solution has been designed to achieve high accuracy, less fault prediction error, and achieve reliability in cloud computing from ML and D-CP techniques.

### 4.3. Acquired Secondary Data

We acquired Antarex HPC fault dataset secondary data through the ZENODO website, and this dataset is published in articles. This dataset and all test environment details are publicly available for use by the community. The Antarex secondary dataset is based on trace data from the homonymous, experimental HPC system at ETH Zurich during fault injection, which is used to undertake ML-based fault prediction studies for researchers.

CPU-Mem mono- has (Instances 4005), CPU-Mem multi- (Instances 4380), HDD mono- (Instances 3244), and HDD multicores (Instances 2493) dataset. This dataset block has eight attributes (timestamp, type, args, seqNum, duration, cores, error, and isFault) and various instances. These instance types are numeric and nominal bases [28]. Table 2 shows a short overview of the secondary dataset.

#### 4.3.1. Exploratory Data Analysis on Secondary Dataset

In the Antarex secondary dataset, we used exploratory data analysis (EDA). The goal of EDA is to tackle specific tasks such as detecting missing and incorrect data, mapping and understanding the underlying structure of the data, and identifying the most important variables in the dataset. The dataset is divided into two sections: CPU and memory-related benchmark apps and fault programs as well as hard-drive-related apps and fault programs. Antarex datasets are organized into four folders: one for each dataset block, namely CPU/memory and HDD, in single-core and multi-core forms [28].

#### 4.3.2. Data Pre-Processing on Secondary Dataset

Data pre-processing is necessary before applying ML algorithms to secondary datasets. This dataset has duplicate values in three attributes named args, seqNum, and duration. Furthermore, this dataset has some none values and empty rows. All duplicate values, none values, and empty rows were removed using the Remove Duplicates option in Excel after applying data pre-processing of the CPU-Mem mono- (Instances 1740), CPU-Mem multi- (Instances 1408), HDD mono- (Instances 568), and HDD multicores (Instances 551).

### 4.4. Generated Primary Data

We generated a primary dataset through the Weibull distribution approach. The Weibull distribution is also often employed as a time-to-failure model for reliability. It extends the exponential model by including non-constant failure rate functions. This contains both rising and falling failure rate curves and has been successfully utilized to explain both initial burnings and wear-out failures [11]. We coded different parameters in the Java platform for primary data generated using the Weibull distribution approach. Table 3 is a summary of the parameters of the primary dataset generated, and Table 4 shows a short overview of the primary dataset. This primary dataset has seven attributes: failure host ID (FHID), host failure time (HFT), last failure time (LFT), distribution (Dis), distribution happen time (DHT), failure time/repair time (FTime/RTime), and status and total (1400) instances. These instance types are numeric and nominal bases.

### 4.5. Data Analysis Techniques

Different ML-based techniques were used in this study for fault classification and prediction. Fault classification and prediction were carried out using various classifiers from NB, LibSVM, MLR, SMO, KNN, and RF algorithms.

#### 4.5.1. Naïve Bayes

**Figure 2:**
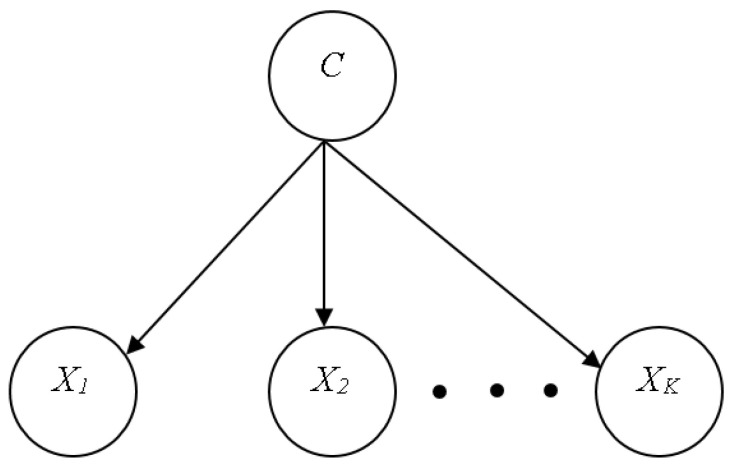
Naïve Bayes Classifier [29].

The NB classifier represents, employs, and learns well-defined probabilistic knowledge. The method is intended for supervised induction tasks where the performance goal is to correctly predict the class of test cases, and the training examples include class information. A naïve classifier is a type of Bayesian network that is based on two basic simplifying assumptions. It assumes, in particular, that the predictive qualities are conditionally independent of the class and that no hidden or latent features influence the prediction process. As a result, Figure 2 depicts the graphic shape of a naïve Bayesian classifier, with all arcs pointing from the class attribute to the observable, predictive attributes [29].

In Equations (2)–(4), the Bayes’ rule is used to compute the probability of each class given a vector of observed values for the predictive qualities and then predicts the most likely class.

(2) $$\begin{array}{c} p\left(C=c|X=x\right)=\frac{p\left(C=c\right)p(X=x|C=c)}{p\left(x=x\right)}\\ p\left(X=x|C=c\right)=p(˄i\;Xi=xi|C=c)\\ =\Pi i\;p\left(Xi=xi|C=c\right) \end{array}$$

Let C represent the random variable representing an instance’s class and X represent a vector of random variables representing the observed attribute values. Let c represent a specific class label and x represent an observed attribute value vector.

(3) p=(X=x C=c)=g(x; μc, σc),where

(4) $$g\left(x;\mu ,\;\sigma \right)=x=\frac{1}{\sqrt{2\pi \sigma }}e-\frac{n\left(x-\sigma \right)²}{2x\sigma ²}$$

We can write the probability density function for a normal (or Gaussian) distribution for continuous attributes.

#### 4.5.2. Library Support Vector Machine

**Figure 3:**
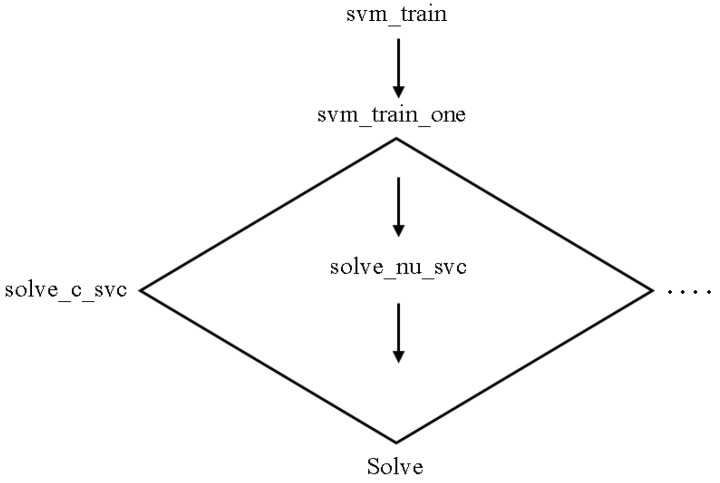
LibSVM Classifier [22].

LIBSVM is an SVM library. The goal is to make applying SVM to applications as simple as possible for users. LIBSVM is widely used in machine learning and a variety of other fields. LIBSVM is commonly used in two steps: first, training a data set to generate a model and then using the model to predict information from a testing data set. LIBSVM provides support for a wide range of SVM formulations for classification, regression, and distribution estimation. Figure 3 depicts the LIBSVM code organization for training [22].

In Equation (5), where e = [1… 1], T is the vector of all ones; Q is a l by l positive semidefinite matrix, Qij yiyjK(xi, xj); and the kernel function is as follows:

(5) K(xi,xj)(xi)T(xj)

#### 4.5.3. Multinomial Logistic Regression

**Figure 4:**
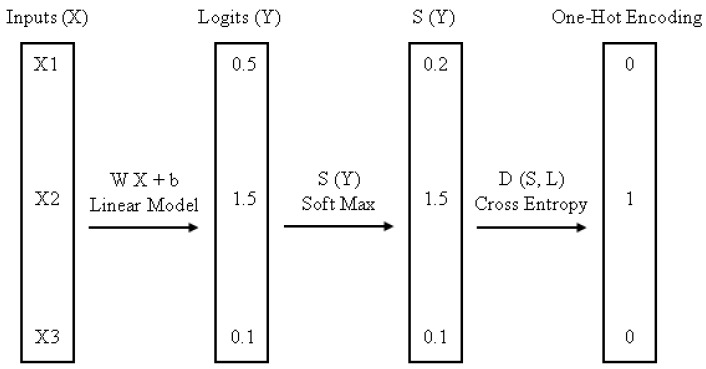
Multinomial Logistic Classifier [31].

Softmax is an abbreviation for MLR. Because of the hypothesis function it employs, regression is a supervised learning technique that can be used to solve a variety of problems, including text categorization. It is a regression model that applies logistic regression to classification problems with multiple possible outcomes [30]. The multinomial logistic classifier is depicted in Figure 4.

(6) $$\begin{array}{c} L_{1}\left(w\right)=\frac{\lambda }{2}\overset{K}{\underset{k=1}{\sum }}||w_{k}|{|}^{2}-\frac{1}{N}\overset{N}{\underset{i=1}{\sum }}\overset{K}{\underset{k=1}{\sum }}y_{ikw_{k}^{T}x_{i}}\\ +\frac{1}{N}\overset{N}{\underset{i=1}{\sum }}log\left(\overset{K}{\underset{k=1}{\sum }}exp\left(w_{k}^{T}x_{i}\right)\right) \end{array}$$

In Equation (6), MLR is employed where the objective function of the classifier is given as above.

#### 4.5.4. Sequential Minimal Optimization

**Figure 5:**
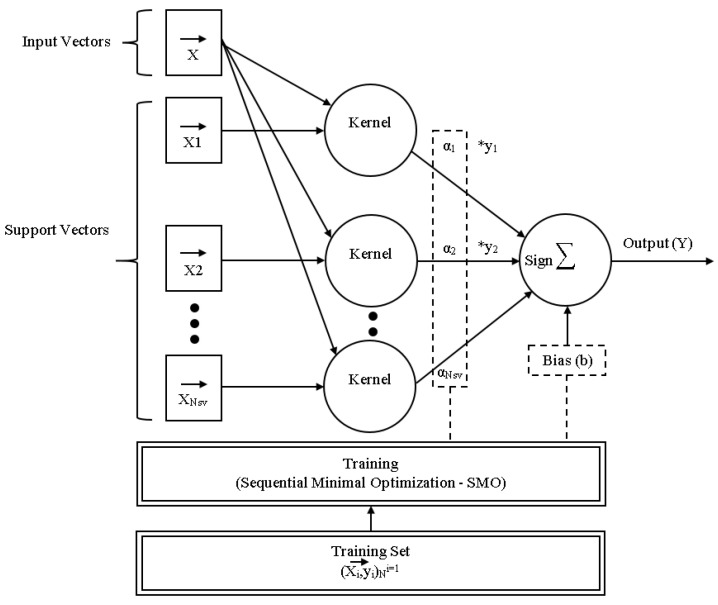
General Architecture of SMO Inference and Training [33].

To train an SVM, a very large quadratic programming (QP) optimization problem must be solved. SMO divides the enormous QP problem into the smallest feasible QP problems. These minor QP issues are handled analytically, which eliminates the need for a time-consuming numerical QP optimization as an inner loop. SMO’s memory requirements scale linearly with training set size, allowing it to handle extremely large training sets. SMO scales the training set size for various test problems somewhere between linear and quadratic because matrix computation is avoided, whereas the traditional chunking SVM technique scales the training set size somewhere between linear and cubic. Because SVM evaluation consumes the majority of SMO’s computing time, SMO is the fastest for linear SVMs and sparse data sets. In real-world sparse data collections, SMO can be more than 1000 times faster than chunking [32]. Figure 5 depicts the overall architecture of SMO inference and training.

In Equations (7)–(9) the QP problem for training an SVM is as given below:

(7) $$w\left(\lambda \right)=\overset{I}{\underset{i=1}{\sum }}\lambda_{i}-\frac{1}{2}\overset{I}{\underset{i=1}{\sum }}\overset{I}{\underset{i=1}{\sum }}yiyjK\left(x_{i}-x_{j}\right)\lambda_{i}\lambda_{j}$$

(8) 0λ−<iC−<,i=1,…,1,

(9) $$\overset{l}{\underset{i=l}{\sum }}y_{i}\lambda_{i}=0$$

In Equation (6), the QP problem for training an SVM is maximized and subject to (7) and (9).

#### 4.5.5. K-Nearest Neighbor

**Figure 6:**
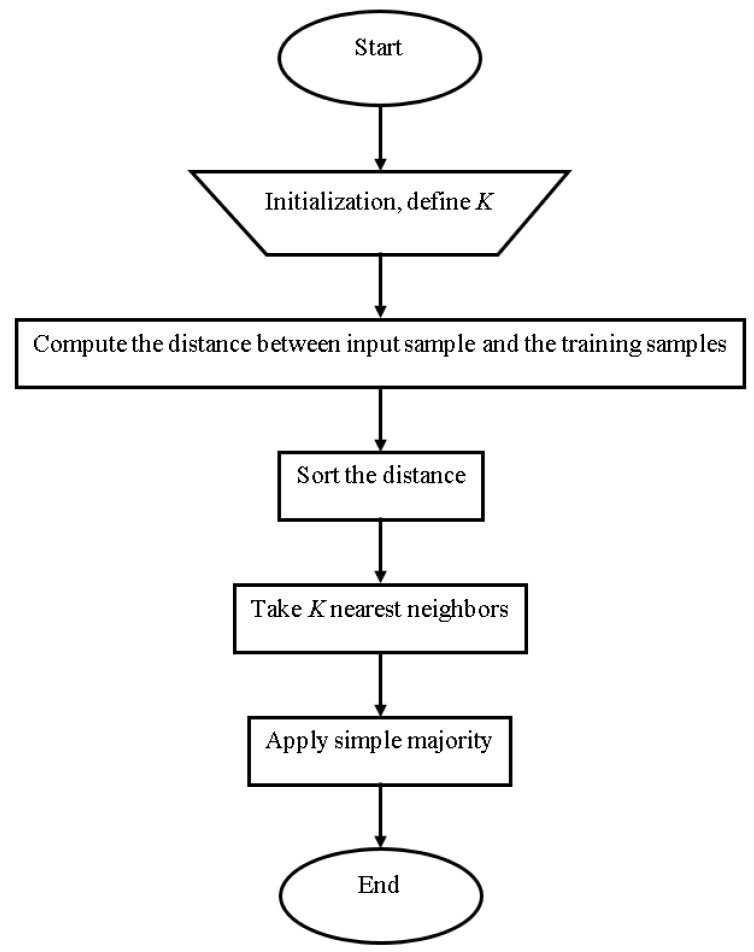
KNN Architecture [35].

The KNN classification method is widely used. It is widely used because of its simplicity and quick calculation time [34]. The choice of value k is critical in this method, as shown in Figure 6. The two parameters that must be accessible to different k values are training and validation error rates [35].

- Determine the parameter K defining the number of nearest neighbors [35];
- Calculate the distance between the query and all training examples [35];
- Using the kth minimum, sort the distance and find the closest neighbors [35];
- Gather the closest neighbors category [35];
- Use the majority in the category of nearest neighbors as the instance’s prediction value [35].

Data are used by fine and medium classifiers to categorize new data points based on similarity measurements.

- Fine and Medium KNN: The fine and medium KNN algorithms use the Euclidean distance function to calculate the nearest neighbors, as shown in Equations (10) and (11).

(10) $$d=\sqrt{\left(x_{1}-y_{1}\right)²+\left(x_{2}-y_{2}\right)²}$$

(11) $$\sqrt{\sum_{i=1}^{k}{\left(x_{i}-y_{i}\right)}^{2}}$$

To calculate the NNs, the fine and medium KNN algorithms employ the Euclidean distance function, as indicated in Equations (10) and (11).

#### 4.5.6. Random Forest

**Figure 7:**
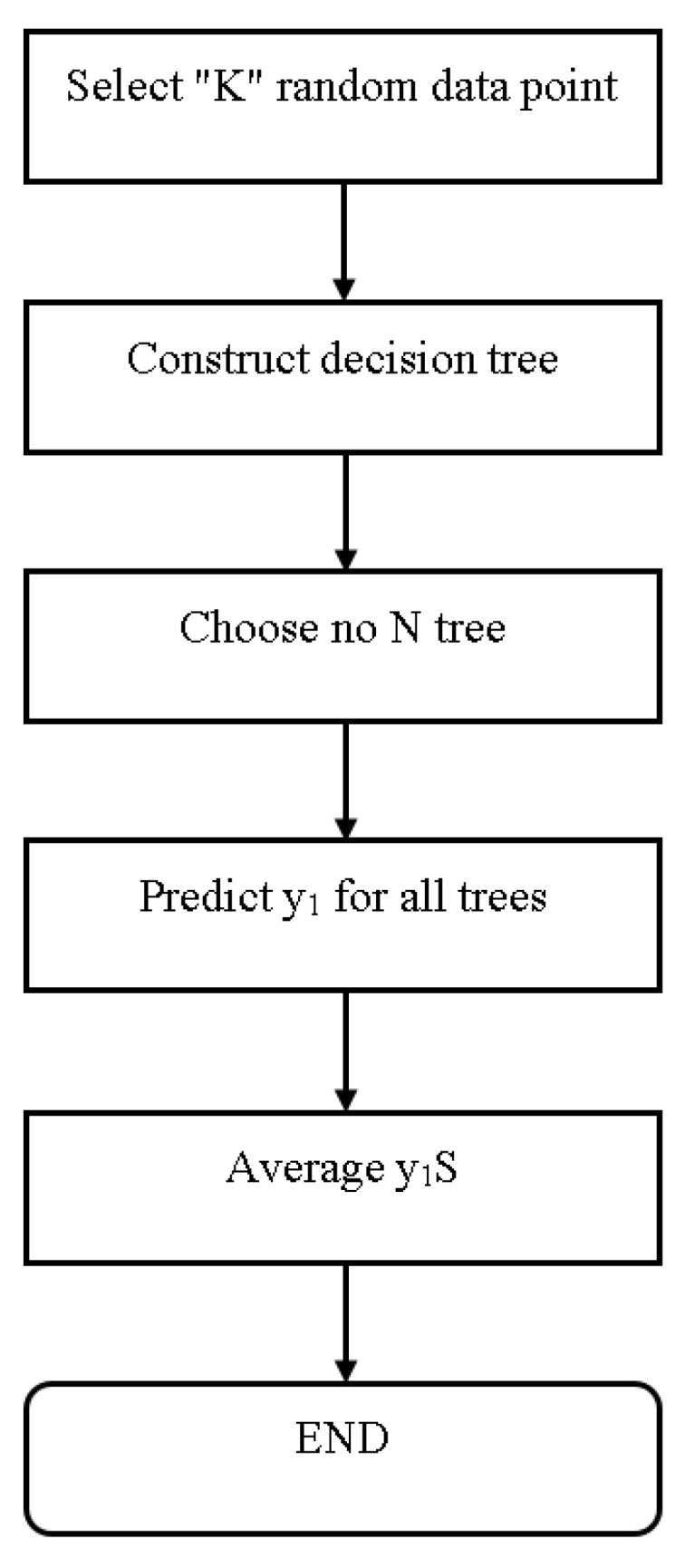
Flowchart of Random Forest Classifier [36].

This method generates a large number of collaborative decision trees. In this algorithm, decision trees serve as pillars. RF is a set of decision trees that were defined during the pre-processing stage. After constructing many trees, the best feature from a random subset of features is chosen. Another idea generated by the decision tree algorithm is the creation of a decision tree. As a result, these trees combine to form a random forest, which is used to classify new objects based on the input vector. Each built decision tree is used to categorize. Figure 7 depicts the flowchart of a random forest classifier [36].

The mathematical formula for RF classifiers is shown below in Equation (12).

(12) $$\begin{array}{c} n_{ij}=w_{i}C_{j}-w_{leftj}C_{leftj}-w_{rightj}C_{rightj}\\ n_{i}\;sub\left(j\right)=\mathrm{the}\;\mathrm{importance}\;\mathrm{of}\;\mathrm{node}\;j\\ w\;sub\left(j\right)=\mathrm{weighted}\;\mathrm{number}\;\mathrm{of}\;\mathrm{samples}\;\mathrm{reaching}\;\mathrm{node}\;j\;\\ C\;sub\left(j\right)=\mathrm{the}\;\mathrm{impurity}\;\mathrm{value}\;\mathrm{of}\;\mathrm{node}\;j\\ left\left(j\right)=\mathrm{child}\;\mathrm{node}\;\mathrm{from}\;\mathrm{left}\;\mathrm{split}\;\mathrm{on}\;\mathrm{node}\;j\;\\ right\left(j\right)=\mathrm{child}\;\mathrm{node}\;\mathrm{from}\;\mathrm{right}\;\mathrm{split}\;\mathrm{on}\;\mathrm{node}\;j \end{array}$$

The mathematical formula for RF classifiers is shown below in (12).

### 4.6. Parameters Configuration of ML Classifiers

ML classifiers have been configured by applying different parameters to achieve accuracy and fault prediction by class. Table 5 shows the different parameters of ML classifiers with values.

### 4.7. Modified Sequential Minimal Optimization

**Figure 8:**
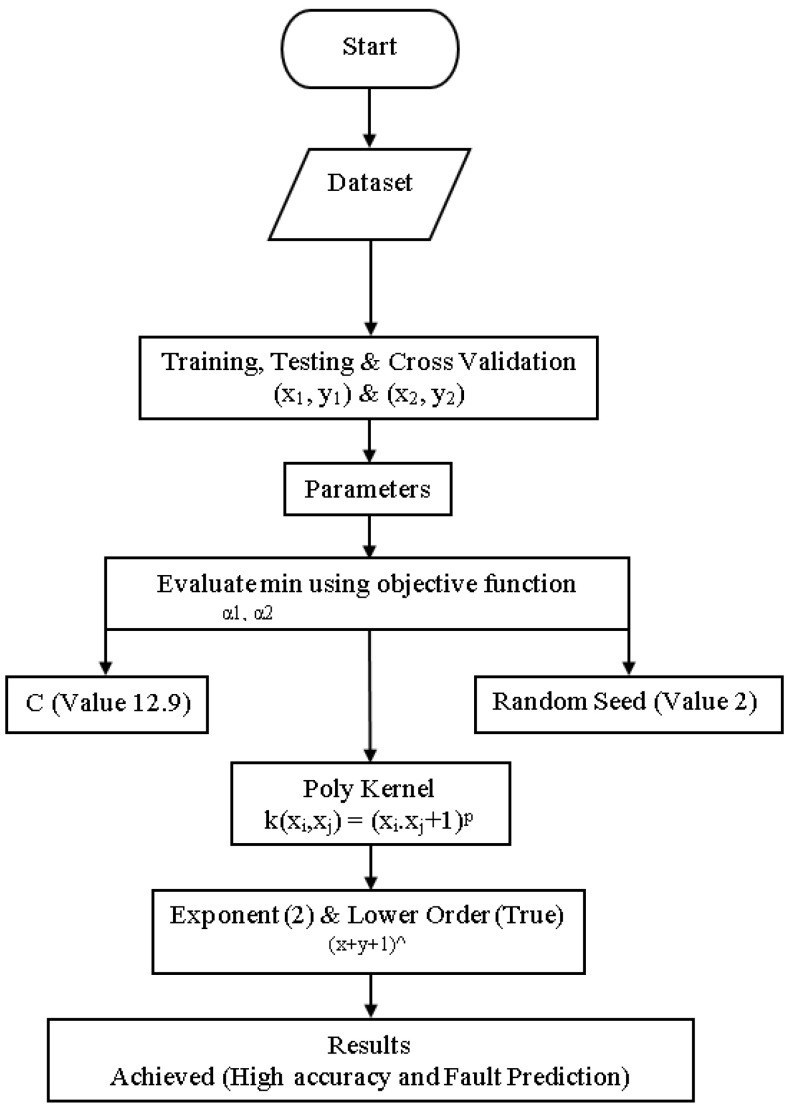
Block Diagram of MSMO Classifier.

The original SMO algorithm has low accuracy and a high fault prediction error. This research has to resolve FT issues by acquiring high accuracy with less fault prediction error to apply to D-CP to achieve reliability by acquiring high accuracy with less fault prediction error from SMO. The block diagram of an MSMO classifier is shown in Figure 8. High accuracy and less fault prediction errors are based on the primary dataset that has been generated. High accuracy and less fault prediction error are evaluated in min α1, α2 using an objective function. High accuracy & less fault prediction error have been made by applying objective functions through algorithm parameters and kernel parameters. The C parameter is determined as a trade-off between fitting the training data and maximizing the separating margin. C has a value between 0.01 and 100. The random seed is set at 2. The only parameter for the polynomial kernel is the exponent, which controls the degree of the polynomial. By default, the kernel computes the exponent as (x*y).

### 4.8. Delta Checkpointing

D-CP is a common basic FT mechanism that works by saving a VMs execution state as an image file regularly. However, due to the limited network resources available in data centers, transferring a large number of CP image files can quickly become congested. This study used a D-CP approach to address this issue, in which the base system is only saved once the first CP is completed, and subsequent CP images only contain incrementally modified pages [9]. The D-CP interval is the amount of time that passes between CPs [37].

#### Description of D-CP Algorithm

The main benefit of using CP is that it allows the cloud computing resources to be used for other customers’ requests while reducing profit loss caused by other methods of fault tolerance. CP interval and latency are two parameters that have a significant impact on the CP algorithm. The CP interval is the amount of time between one CP and the next. CP latency is the amount of time it takes to save a CP.

The CP algorithm assumes that the length of the CP interval must not be fixed while the customer’s task is being executed. At the time of the current CP, the algorithm calculates the next CP interval. This is determined by the failed history of the VM on which the task is run. If the failure history is poor, the algorithm will shorten the CP interval. Furthermore, if the failure history is good, the algorithm will extend the CP interval. Equations (13)–(18) values are based on the D-CP algorithm.

τ_ji_ (13)

The execution time of task *j* on VM *i.*

τϒ_ji_ (14)

The remaining execution time of task *j* on VM *i.*

*F*_*i*_(*x*_1_) (15)

Failure probability of VM *i.*

*Fi*(*x*_0_) (16)

Probability of no failure of VM *i.*

*h* (17)

CP interval.

z(18)

Number of failures during the task execution.

### 4.9. Reliability

The dependability of each VM will be assessed, and cloudlets will be assigned to the most reliable VM. It is the cloud broker’s responsibility to assign cloudlets to cloud providers. To evaluate VM reliability, we first determine whether cloudlets executed successfully or failed within the time limit. Then, we update the reliability of each VM based on success and failure cloudlets. Finally, we select the most dependable VM from the list of available VMs and assign cloudlets to it [38].

In the equation, (19) represents the VM’s reliability, and (20) to (21) represents the host’s reliability, where MMi is the available memory ratio, CPi is the available MIPS ratio, BWi is the available bandwidth ratio, and Ri is the reliability of the ith VM.

(19) $$\frac{\Sigma \mathrm{cloudleti}\;\mathrm{RAMi}+\mathrm{CPUi}+\mathrm{BWi}}{\mathrm{No}.\;\mathrm{of}\;\mathrm{Cloudlet}}$$

Reliability of VM.

(20) $$\mathrm{MMi}=\frac{\mathrm{Available}\;\mathrm{RAMi}}{\mathrm{Total}\;\mathrm{RAM}}$$

Now, (20) is used to calculate the VMs available RAM ratio.

(21) $$\mathrm{Ri}=\frac{\mathrm{CPUi}+\mathrm{MMi}+\mathrm{BWi}}{3}$$

(21) The Ri reliability of ith in the VM is found.

Similarly, as shown in Equations steps (22)–(24), the availability of MIPS ratio CPi and bandwidth ratio BWi can be calculated as the ratio of available MIPS to total MIPS and available bandwidth to total bandwidth.

(22) $$\frac{{\Sigma \mathrm{vmi}}^{\mathrm{reliability}}\mathrm{vmi}}{3}$$

Reliability of host.

(23) $${\mathrm{CP}}_{i}=\frac{\mathrm{Available}\;\mathrm{MIPSi}}{\mathrm{Total}\;\mathrm{MIPS}}$$

Next, (23) is used to obtain the ratio of available MIPS in the VM.

(24) $${\mathrm{BW}}_{i}=\frac{\mathrm{Available}\;\mathrm{BWi}}{\mathrm{Total}\;\mathrm{BW}}$$

Then, (24) is used to find the BWi available bandwidth ratio in the VM.

## 5. Results and Findings

Data analysis is performed in this section. Classification results using NB, LibSVM, MLR, SMO, and RF with confusion matrix and graphical representations results are incorporated into this section. Finally, MSMO results, which are the main algorithm of this research study, are also included here. This research focuses on a comparative analysis of conventional, ML algorithms, and FT techniques for high accuracy, less fault prediction error, and reliability.

The secondary dataset archive includes four directories: one for each dataset block, namely CPU/memory and HDD, in single-core and multi-core variants [10]. A significant difference was observed in the four directories of the secondary dataset based on results, the difference is CPU-Mem multicores have good results against the remaining directories such as CPU-Mem mono, HDD mono, and HDD multi.

According to the comparisons, the primary dataset has good results against the secondary dataset, so in this research, the primary dataset results were sufficient to consider in terms of modification of the ML algorithm.

ML classifiers were used before using the FT Delta-CP approach. Data were trained on 80/20, 70/30, and 5-fold cross-validation using NB, LibSVM, MLR, SMO, KNN, and RF classifiers, and the desired results in the classification (secondary and primary) were achieved. The results are compared based on NB, LibSVM, MLR, SMO, KNN, and RF in terms of accuracy, fault prediction error, and data validation by class using the following Equations (25)–(36). Secondary dataset (CPU-Mem multi) results proved that NB outperformed LibSVM, MLR, SMO, KNN, and RF. Furthermore, the primary dataset results proved that RF outperformed, but the time complexity was not good. According to the primary dataset results, RF and SMO have minor point values difference between results, but SMO yielded good time complexity. The software environment we used is WEKA 3.8.6 with Remove Percentage Filter.

(25) $$\mathrm{Accuracy}=\frac{\mathrm{TP}+\mathrm{TN}}{\mathrm{TP}+\mathrm{TN}+\mathrm{FP}+\mathrm{FN}}$$

In Equation (25), the accuracy is defined as above.

(26) $$\mathrm{Recall}\;\mathrm{or}\;\mathrm{True}-\mathrm{Positive}\;\mathrm{Rate}=\frac{\mathrm{TP}}{\mathrm{TP}+\mathrm{FN}}$$

In Equation (26), the recall or true-positive rate is defined as above.

(27) $$\mathrm{True}-\mathrm{Negative}\;\mathrm{Rate}=\frac{\mathrm{TN}}{\mathrm{TN}+\mathrm{FP}}$$

In Equation (27), the true-negative rate is defined as above.

(28) $$\mathrm{Precision}=\frac{\mathrm{TP}\;}{\mathrm{TP}+\mathrm{FP}}$$

In Equation (28), the precision is defined as above.

(29) $$\mathrm{False}-\mathrm{Positive}\;\mathrm{Rate}=\frac{\mathrm{FP}\;}{\mathrm{TN}+\mathrm{FP}}$$

In Equation (29), the false-positive rate is defined as above.

(30) $$\mathrm{MCC}=\frac{\mathrm{TP}.\mathrm{TN}\;-\;\mathrm{FP}.\mathrm{FN}\;}{\surd \left(\mathrm{TP}+\mathrm{FP}\right)\left(\mathrm{TP}+\mathrm{FN}\right)\left(\mathrm{TN}+\mathrm{FP}\right)\left(\mathrm{TN}+\mathrm{FN}\right)}$$

In Equation (30), the Matthews correlation coefficient is defined as above.

(31) $$F-\mathrm{Measure}=\frac{2\mathrm{PPV}\;\times \;\mathrm{TPR}\;}{\mathrm{PPV}+\mathrm{TPR}}$$

In Equation (31), the F-measure is defined as above.

(32) $$F1\;\mathrm{Score}=\frac{2*\mathrm{Precision}*\mathrm{Recall}\;}{\mathrm{Precision}+\mathrm{Recall}}$$

In Equation (32), the F1 score is defined as above.

- The RMSE is a commonly used measure of the difference between predicted and observed values by a model or estimator [39];
- MAE is a distinct measure of two continuous variables [39];
- The relative absolute error normalizes the total absolute error by dividing it by the total absolute error of the simple predictor [40];
- The relative squared error normalizes the total squared error by dividing it by the simple predictor’s total squared error [40].

(33) $$\mathrm{RMSE}=\sqrt{\frac{1}{n}\sum_{i=1}^{n}{\left(y_{i}-\hat{yi}\right)}^{2}}$$

In Equation (33), the RMSE is defined as follows.

(34) $$\mathrm{MAE}=\frac{1}{n}\overset{n}{\underset{i=1}{\sum }}\left|y_{i}-\hat{y_{i}}\right|$$

In Equation (34), the MAE is defined as follows.

(35) $$E_{i}=\frac{\sum_{j=1}^{n}\left|P_{\left(ij\right)}-T_{j}\right|}{\sum_{j=1}^{n}\left|T_{j}-\bar{T}\right|}$$

In Equation (35), the RAE is defined as follows.

(36) $$E_{i}=\sqrt{\frac{\sum_{j=1}^{n}{\left(P_{\left(ij\right)}-T_{j}\right)}^{2}}{\sum_{j=1}^{n}{\left(T_{j}-\bar{T}\right)}^{2}}}$$

In Equation (36), the RSE is defined as follows.

### 5.1. Simulation Setup of ML Classifiers to Achieve High Accuracy and Less Fault Prediction

WEKA stands for Waikato environment for knowledge analysis and refers to software written in Java by the University of Waikato in New Zealand and distributed under the GNU general public license. This software consists of a collection of [41] machine learning algorithms [42] and data pre-processing and transformation tools, including discretization and sampling methods [41]. Table 6 shows the configuration description for an experiment.

### 5.2. Comparison of Classification Models on Secondary Dataset

We present the results associated with different classifiers using ISFAULT class in the secondary dataset. For classification models, we opted for NB, LibSVM, MLR, RF, KNN, and SMO with the poly kernel.

The secondary data results of each classifier are shown in Figure 9, Figure 10, Figure 11, Figure 12, Figure 13, Figure 14, Figure 15, Figure 16, Figure 17, Figure 18, Figure 19, Figure 20, Figure 21, Figure 22, Figure 23, Figure 24, Figure 25, Figure 26, Figure 27, Figure 28, Figure 29, Figure 30, Figure 31, Figure 32, Figure 33, Figure 34, Figure 35, Figure 36, Figure 37, Figure 38, Figure 39, Figure 40, Figure 41, Figure 42, Figure 43, Figure 44, Figure 45, Figure 46, Figure 47, Figure 48, Figure 49, Figure 50, Figure 51, Figure 52, Figure 53, Figure 54, Figure 55, Figure 56, Figure 57, Figure 58, Figure 59, Figure 60, Figure 61, Figure 62, Figure 63, Figure 64, Figure 65, Figure 66, Figure 67, Figure 68, Figure 69, Figure 70, Figure 71 and Figure 72, with the 80/20, 70/30, and 5-fold cross-validation in terms of high accuracy and less fault prediction. Furthermore, data validation was 60% training, 20% testing, and 20% validation. In the secondary data results, CPU-Mem mono gave the highest percentage of accuracy and less fault prediction to the NB classifier in terms of 80/20 (77.01%), 70/30 (76.05%), and 5 -old cross-validation (74.88%) and CPU-Mem multi in terms of 80/20 (89.72%), 70/30 (90.28%), and 5-fold cross-validation (92.83%). Furthermore, for HDD mono, the SMO classifier gave the highest percentage of accuracy and less fault prediction fault in terms of 80/20 (87.72%), 70/30 (89.41%), and 5-fold cross-validation (88.38%) and HDD-multi in terms of 80/20 (93.64%), 70/30 (90.91%), and 5-fold cross-validation (88.20%). According to the results, the difference is that CPU-Mem multicores have good results against the remaining directories of CPU-Mem mono, HDD mono, and HDD multi.

#### 5.2.1. Secondary Dataset CPU-Mem Mono Block-I

**Figure 9:**
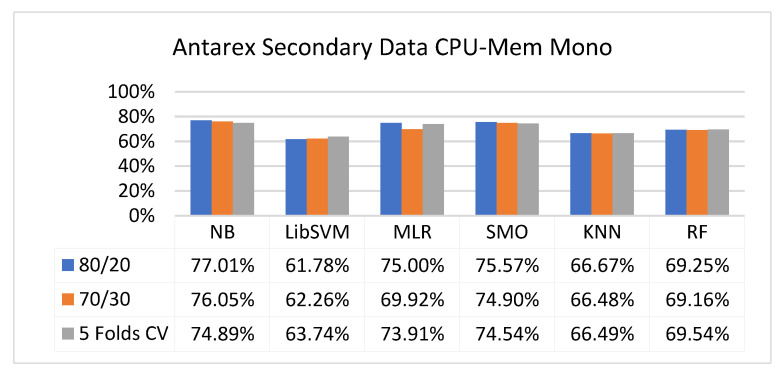
Accuracy by Class (True/False) of CPU-Mem Mono on ML Classifiers.

**Figure 10:**
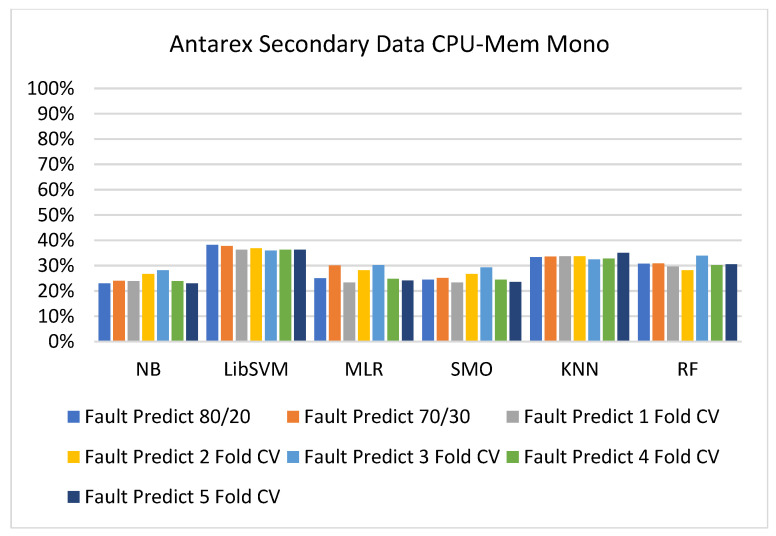
Fault Prediction by Class (True/False) of CPU-Mem Mono on ML Classifiers.

**Figure 11:**
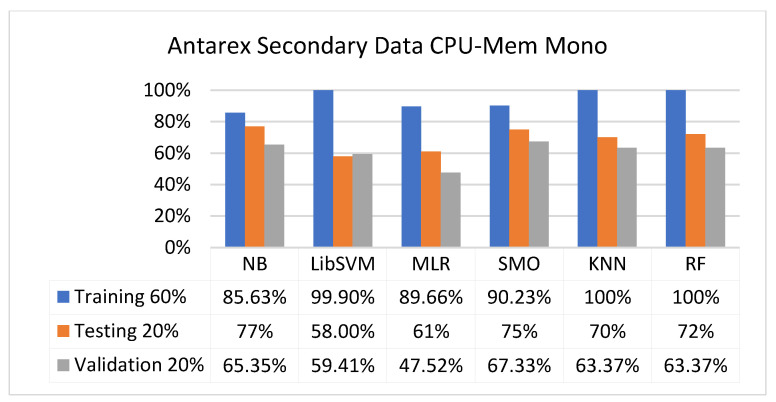
Accuracy by Class (True/False) of CPU-Mem Mono on ML Classifiers Related to Data Validation Results.

**Figure 12:**
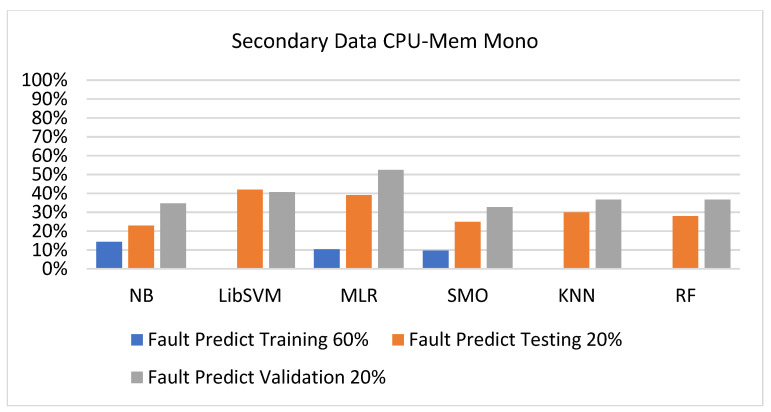
Fault Prediction by Class (True/False) of CPU-Mem Mono on ML Classifiers Related to Data Validation Results.

Figure 9, Figure 10, Figure 11 and Figure 12 depict a comparison of the results of NB, LibSVM, MLR, SMO, KNN, and RF in CPU-Mem-mono-related detailed accuracy by class (true/false) and prediction on test-split additional data validation.

**Figure 13:**
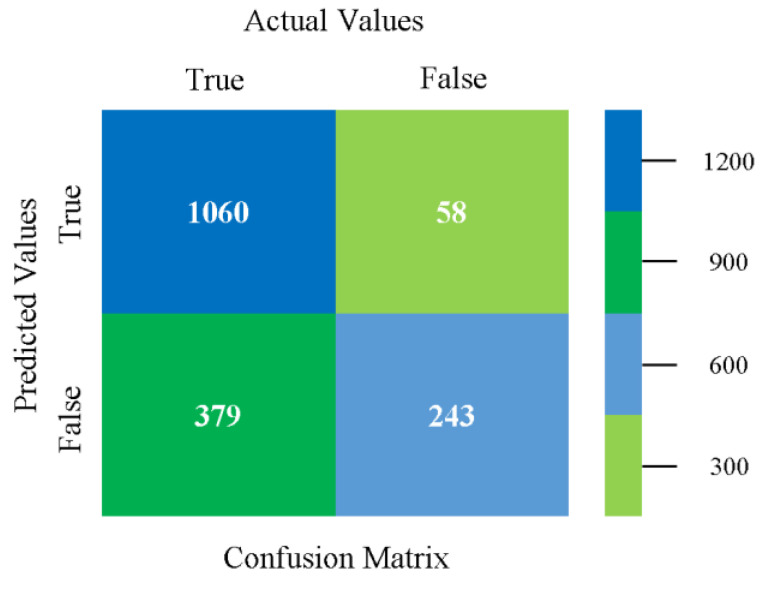
Confusion Matrix of NB Classifier based on CPU-Mem Mono in Accuracy and Fault Prediction.

**Figure 14:**
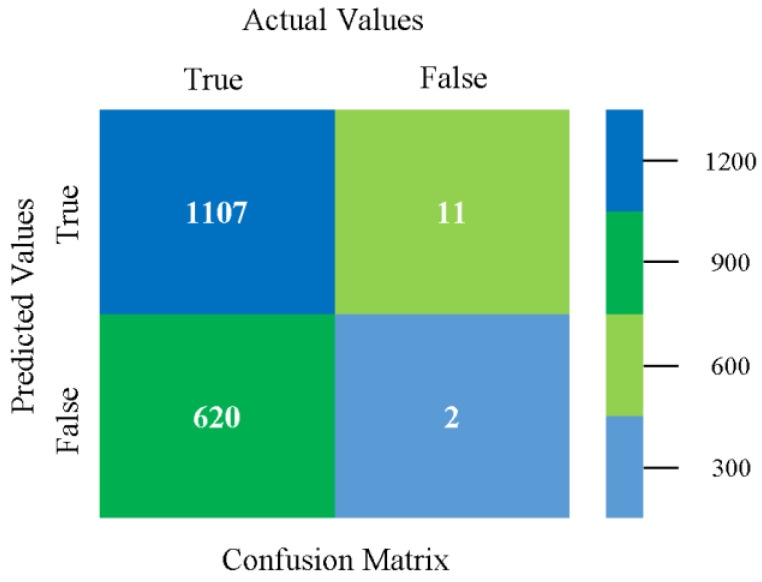
Confusion Matrix of LibSVM Classifier based on CPU-Mem Mono in Accuracy and Fault Prediction.

**Figure 15:**
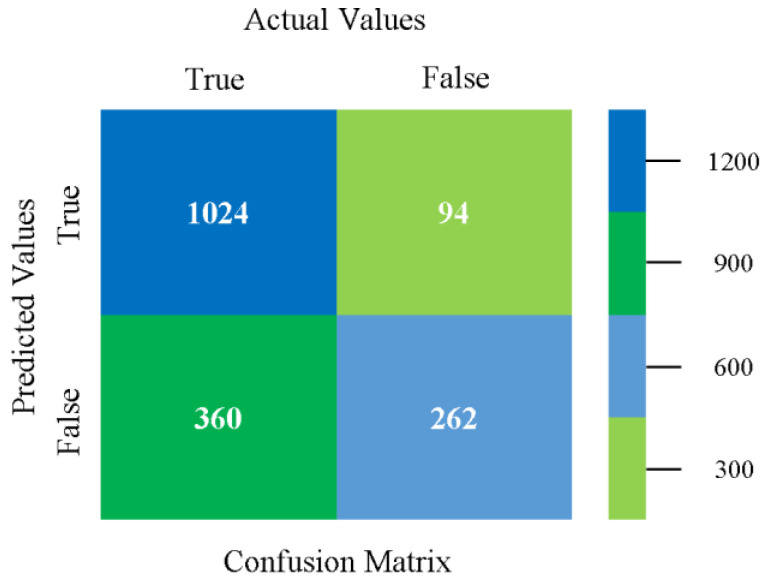
Confusion Matrix of MLR Classifier based on CPU-Mem Mono in Accuracy and Fault Prediction.

**Figure 16:**
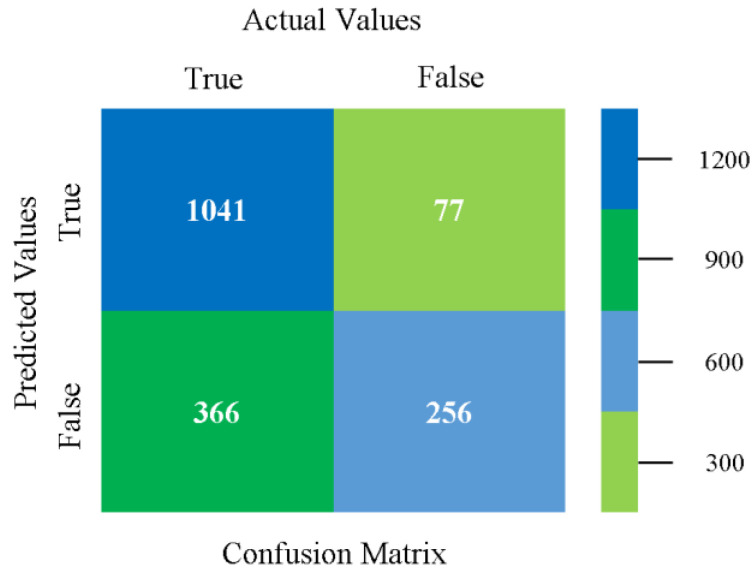
Confusion Matrix of SMO Classifier based on CPU-Mem Mono in Accuracy and Fault Prediction.

**Figure 17:**
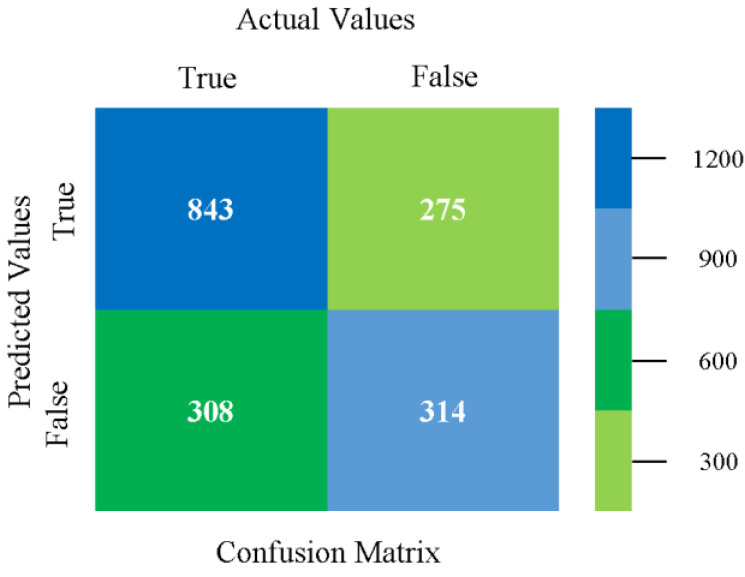
Confusion Matrix of KNN Classifier based on CPU-Mem Mono in Accuracy and Fault Prediction.

**Figure 18:**
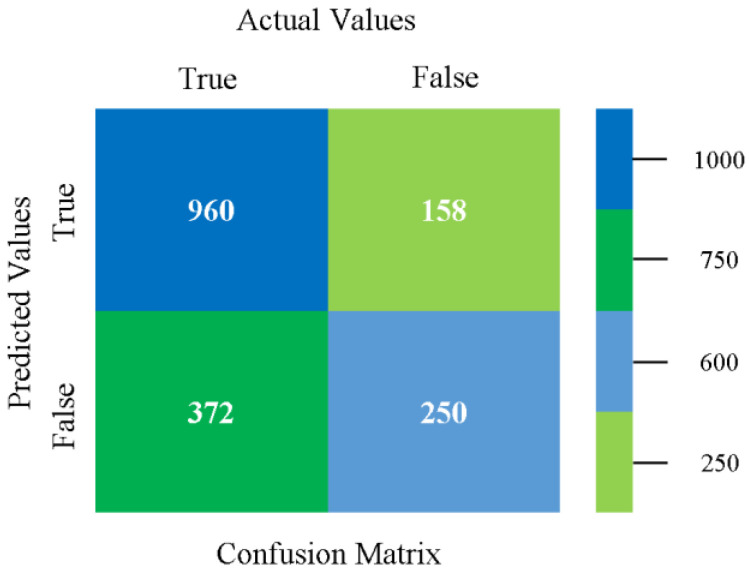
Confusion Matrix of RF Classifier based on CPU-Mem Mono in Accuracy and Fault Prediction.

The confusion matrix is used to calculate accuracy, precision, recall, and F-measure. It is used as an efficient technique for the classification of attributes based on qualitative response categories. Figure 13, Figure 14, Figure 15, Figure 16, Figure 17 and Figure 18 show the confusion matrix related to accuracy and fault prediction achieved through NB, LibSVM, MLR, SMO, KNN, and RF. The following confusion matrix indicates that the NB classification model gave the highest percentage of accuracy and less fault prediction for CPU-Mem mono.

**Figure 19:**
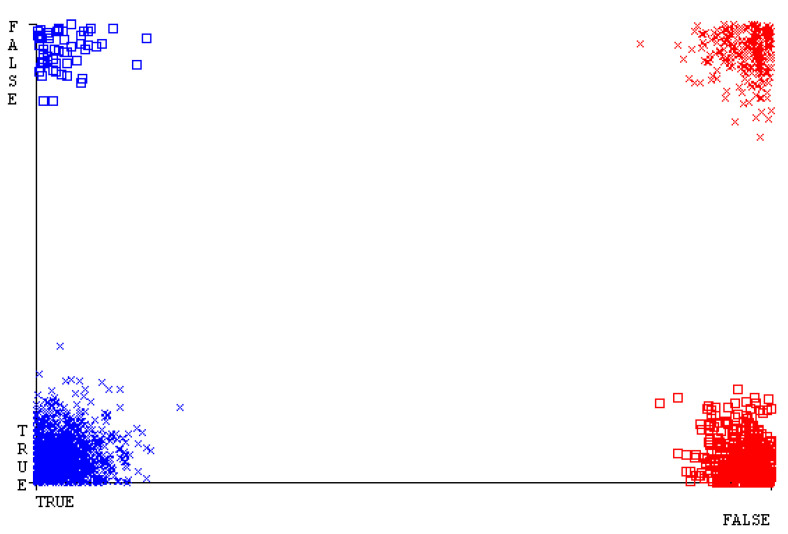
Classifier Errors of NB Classifier based on CPU-Mem Mono in Accuracy and Fault Prediction.

**Figure 20:**
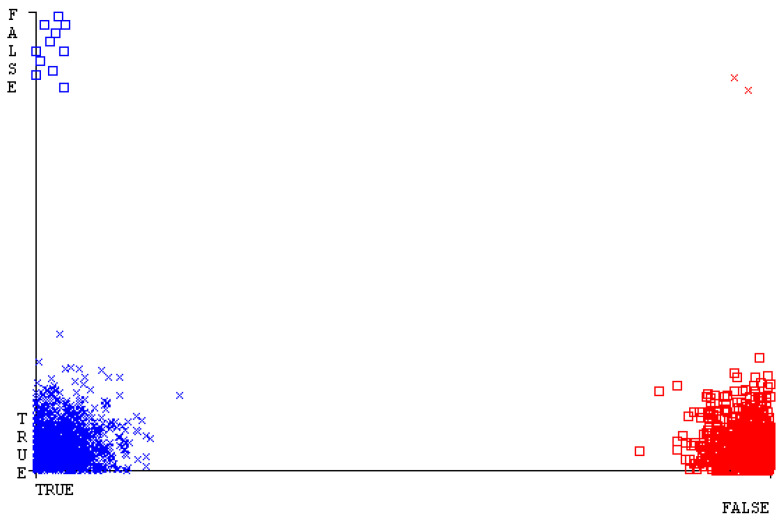
Classifier Errors of LibSVM Classifier based on CPU-Mem Mono in Accuracy and Fault Prediction.

**Figure 21:**
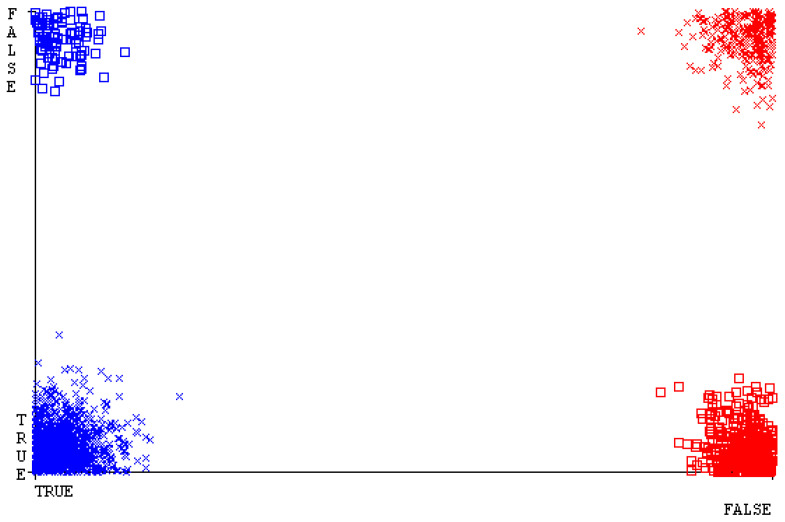
Classifier Errors of MLR Classifier based on CPU-Mem Mono in Accuracy and Fault Prediction.

**Figure 22:**
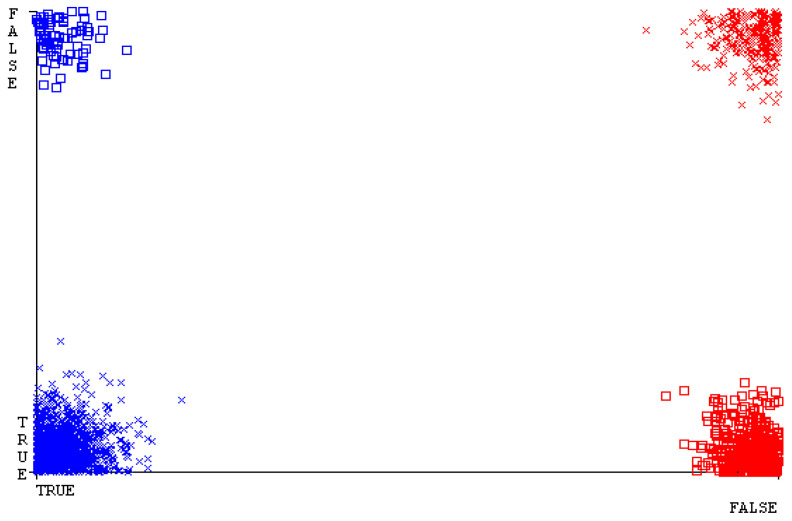
Classifier Errors of SMO Classifier based on CPU-Mem Mono in Accuracy and Fault Prediction.

**Figure 23:**
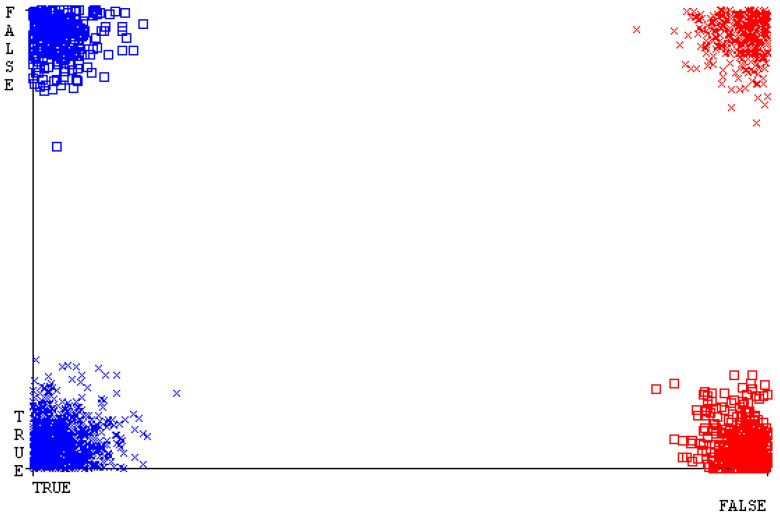
Classifier Errors of KNN Classifier based on CPU-Mem Mono in Accuracy and Fault Prediction.

**Figure 24:**
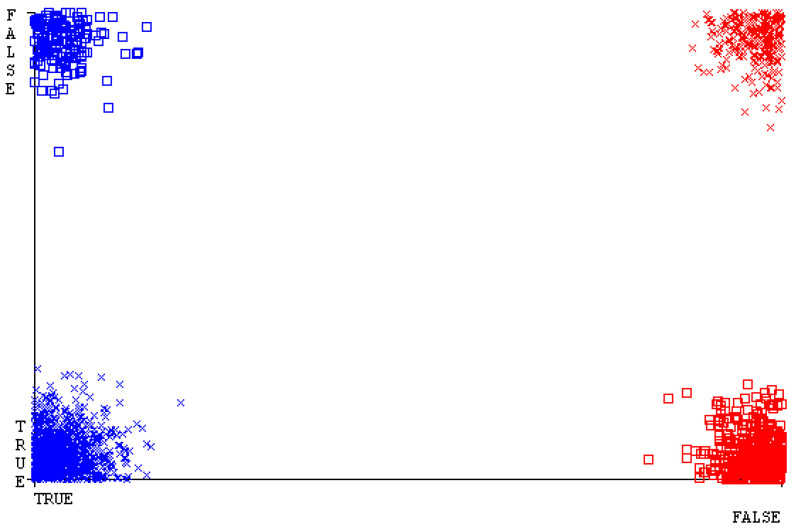
Classifier Errors of RF Classifier based on CPU-Mem Mono in Accuracy and Fault Prediction.

Figure 19, Figure 20, Figure 21, Figure 22, Figure 23 and Figure 24 represent the error of the classifier that shows the values corresponding to true-positive, true-negative, false-positive, and false-negative values. In Figure 19, Figure 20, Figure 21, Figure 22, Figure 23 and Figure 24, the square box represents the errors in the actual class versus the predicted class.

#### 5.2.2. Secondary Dataset CPU-Mem Multi Block-II

**Figure 25:**
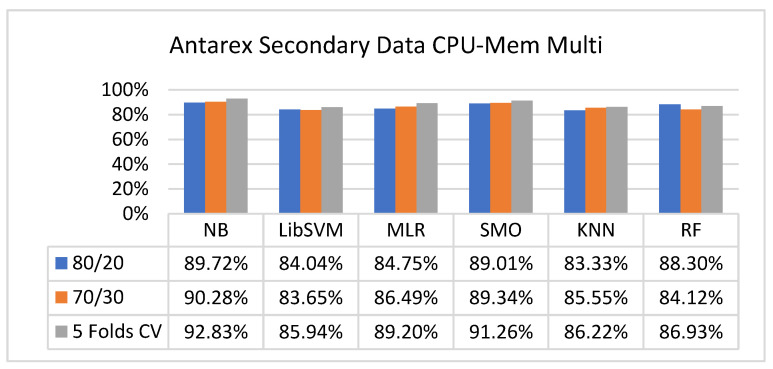
Accuracy by Class (True/False) of CPU-Mem Multi on ML Classifiers.

**Figure 26:**
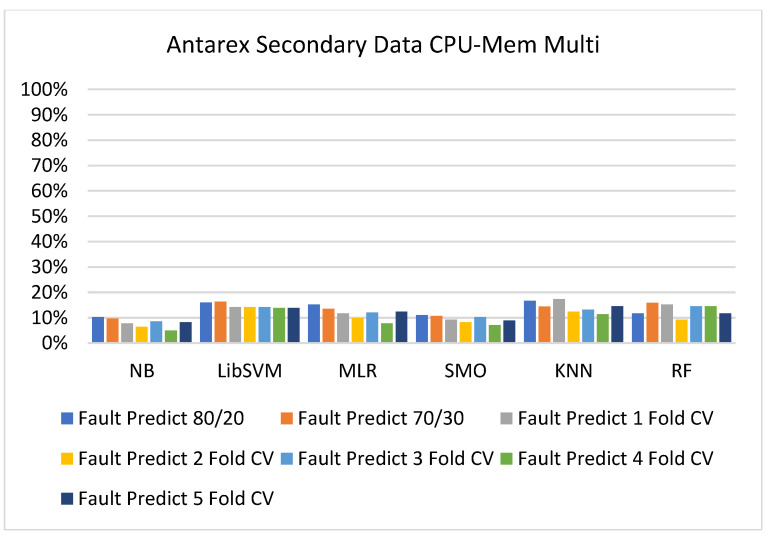
Fault Prediction by Class (True/False) of CPU-Mem Multi on ML Classifiers.

**Figure 27:**
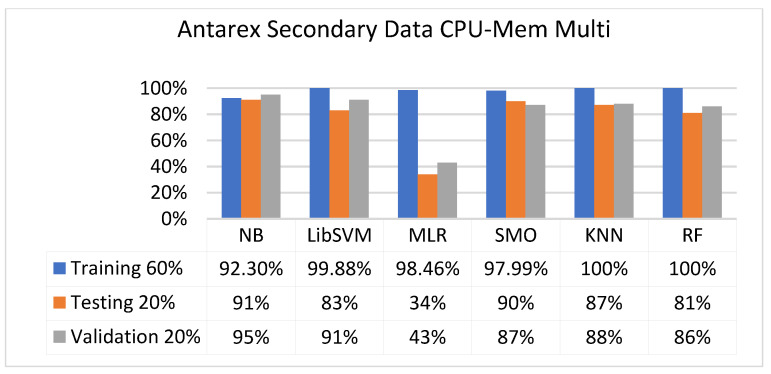
Accuracy by Class (True/False) of CPU-Mem Multi on ML Classifiers Related to Data Validation Results.

**Figure 28:**
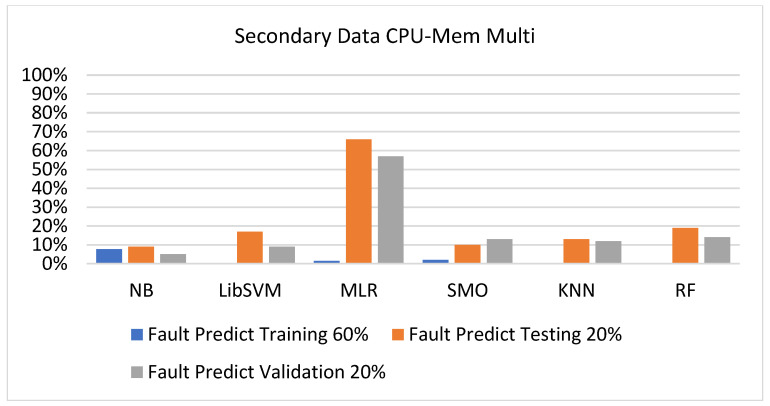
Fault Prediction by Class (True/False) of CPU-Mem Multi on ML Classifiers Related to Data Validation Results.

Figure 25, Figure 26, Figure 27 and Figure 28 depict a comparison of the results of NB, LibSVM, MLR, SMO, KNN, and RF in CPU-Mem multi related to detailed accuracy by class (true/false) and prediction on test-split additional data validation.

**Figure 29:**
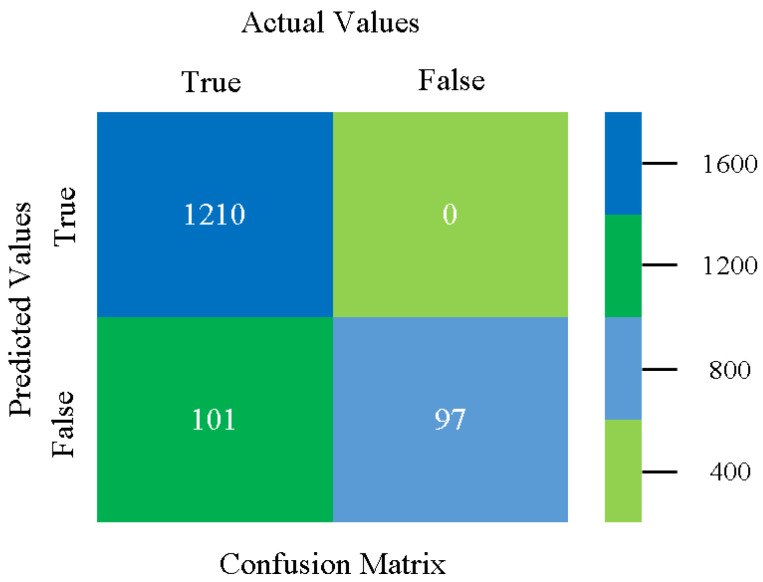
Confusion Matrix of NB Classifier based on CPU-Mem Multi in Accuracy and Fault Prediction.

**Figure 30:**
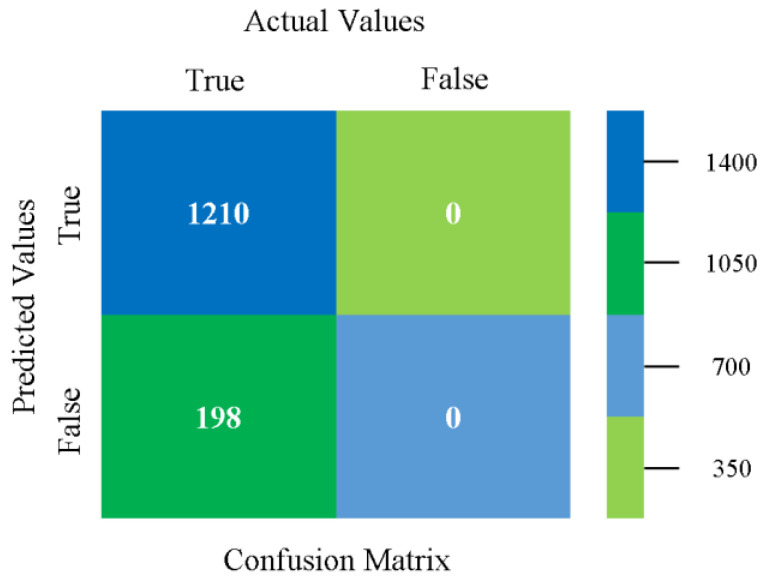
Confusion Matrix of LibSVM Classifier based on CPU-Mem Multi in Accuracy and Fault Prediction.

**Figure 31:**
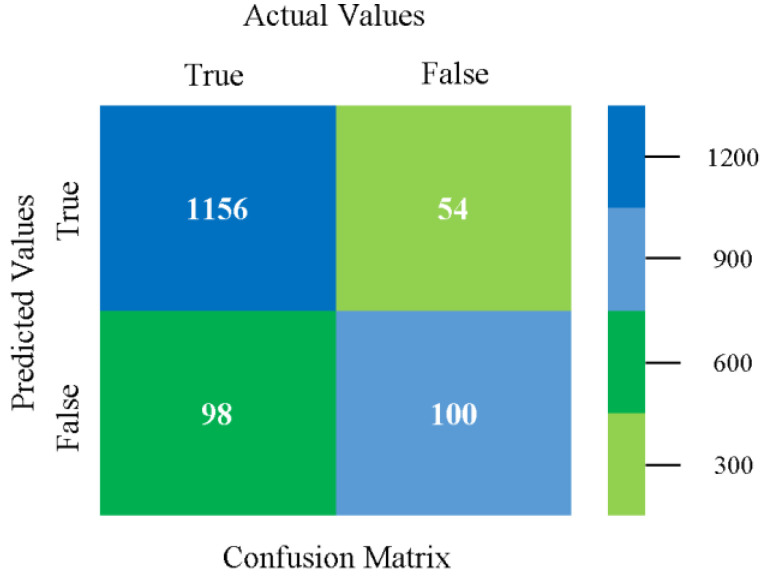
Confusion Matrix of MLR Classifier based on CPU-Mem Multi in Accuracy and Fault Prediction.

**Figure 32:**
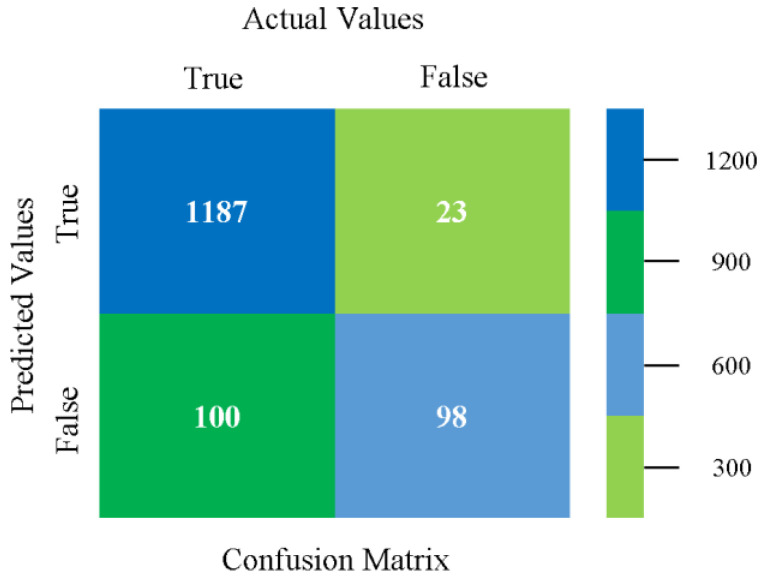
Confusion Matrix of SMO Classifier based on CPU-Mem Multi in Accuracy and Fault Prediction.

**Figure 33:**
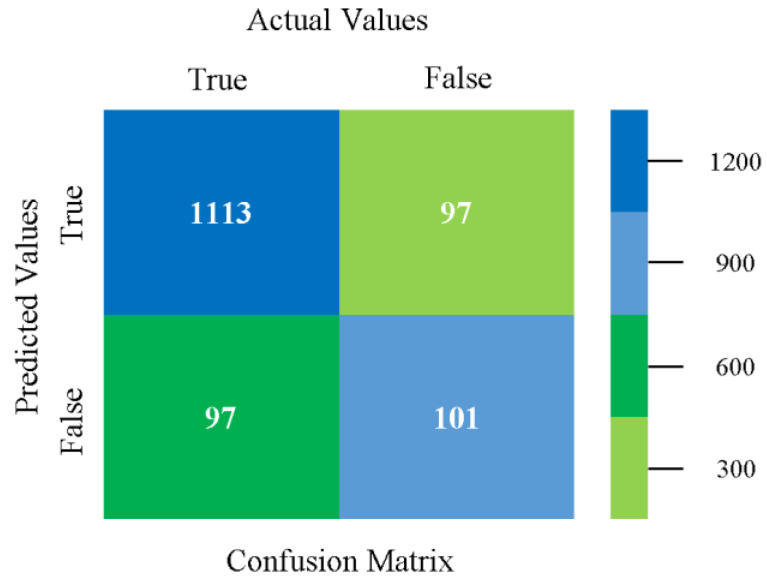
Confusion Matrix of KNN Classifier based on CPU-Mem Multi in Accuracy and Fault Prediction.

**Figure 34:**
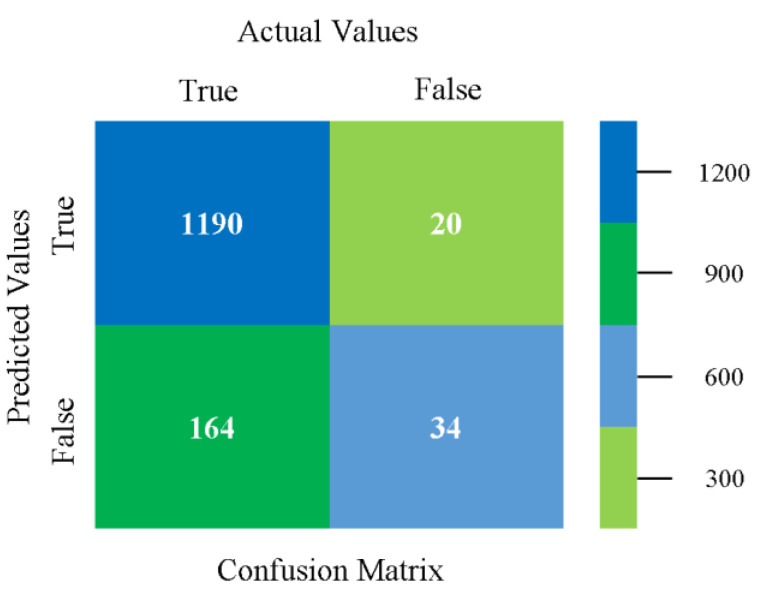
Confusion Matrix of RF Classifier based on CPU-Mem Multi in Accuracy and Fault Prediction.

The confusion matrix is used to calculate accuracy, precision, recall, and F-measure. It is used as an efficient technique for the classification of attributes based on qualitative response categories. Figure 29, Figure 30, Figure 31, Figure 32, Figure 33 and Figure 34 show the confusion matrix related to accuracy and fault prediction achieved through NB, LibSVM, MLR, SMO, KNN, and RF. The following confusion matrix indicates that the NB classification model gave the highest percentage of accuracy and less fault prediction for CPU-Mem multi.

**Figure 35:**
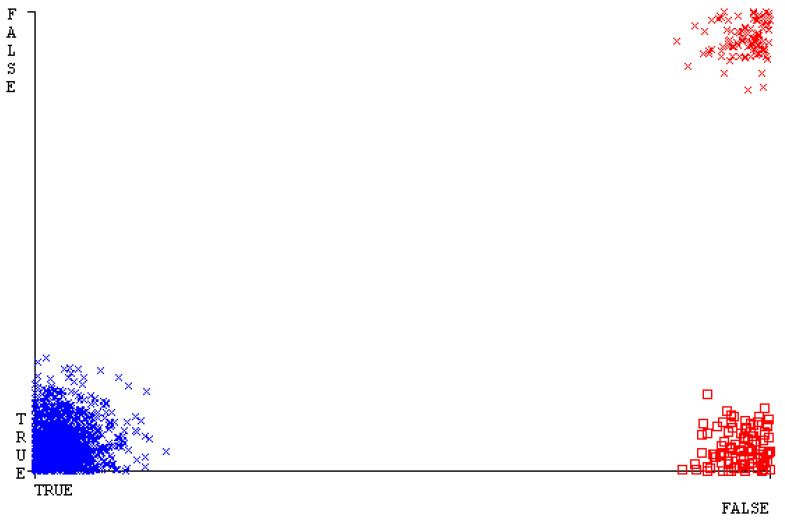
Classifier Errors of NB Classifier based on CPU-Mem Multi in Accuracy and Fault Prediction.

**Figure 36:**
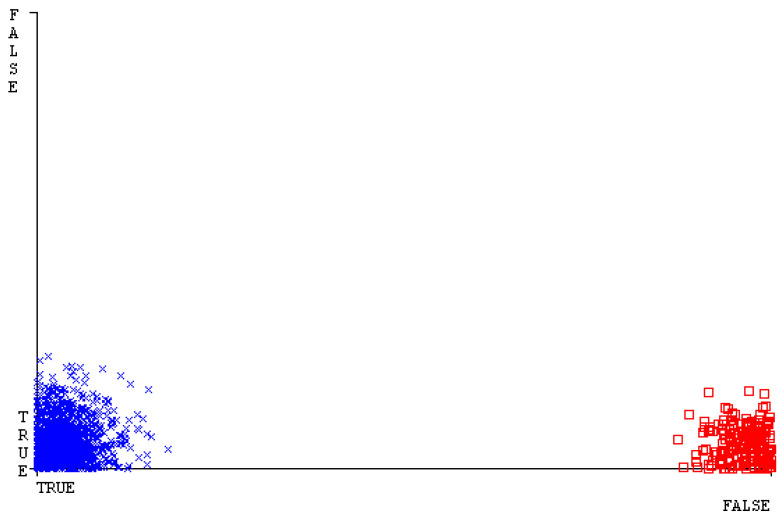
Classifier Errors of LibSVM Classifier based on CPU-Mem Multi in Accuracy and Fault Prediction.

**Figure 37:**
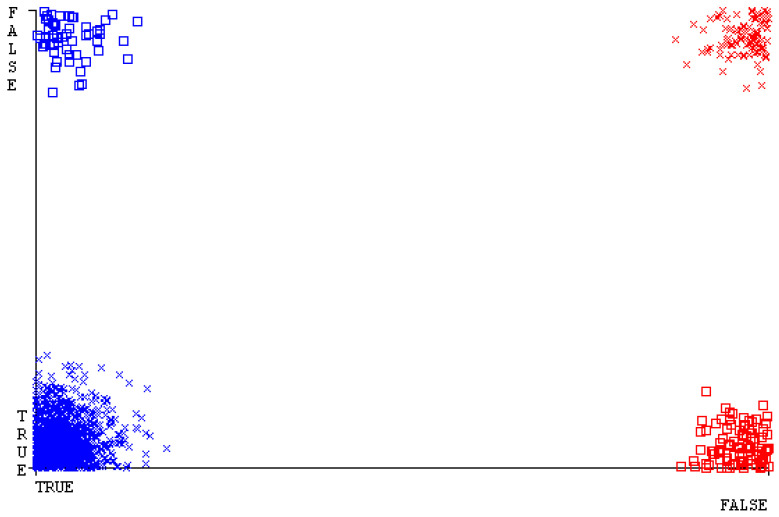
Classifier Errors of MLR Classifier based on CPU-Mem Multi in Accuracy and Fault Prediction.

**Figure 38:**
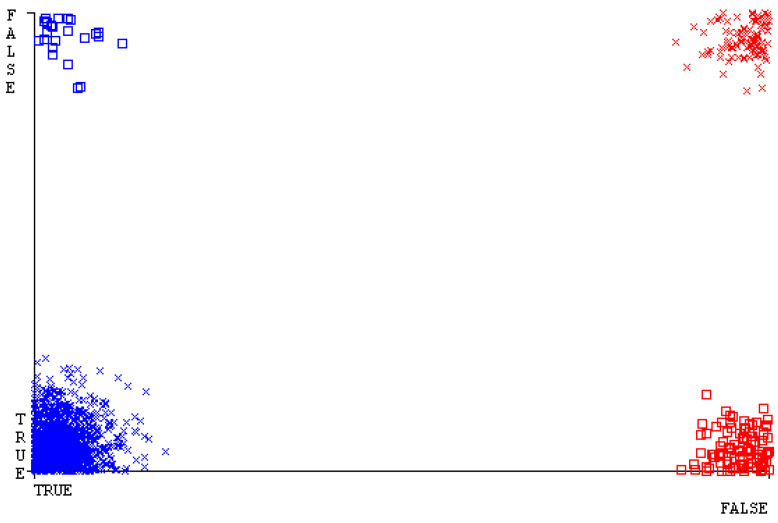
Classifier Errors of SMO Classifier based on CPU-Mem Multi in Accuracy and Fault Prediction.

**Figure 39:**
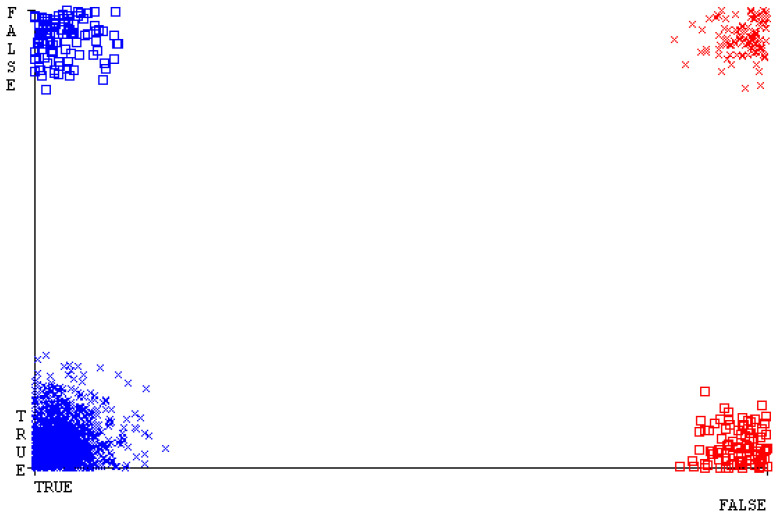
Classifier Errors of KNN Classifier based on CPU-Mem Multi in Accuracy and Fault Prediction.

**Figure 40:**
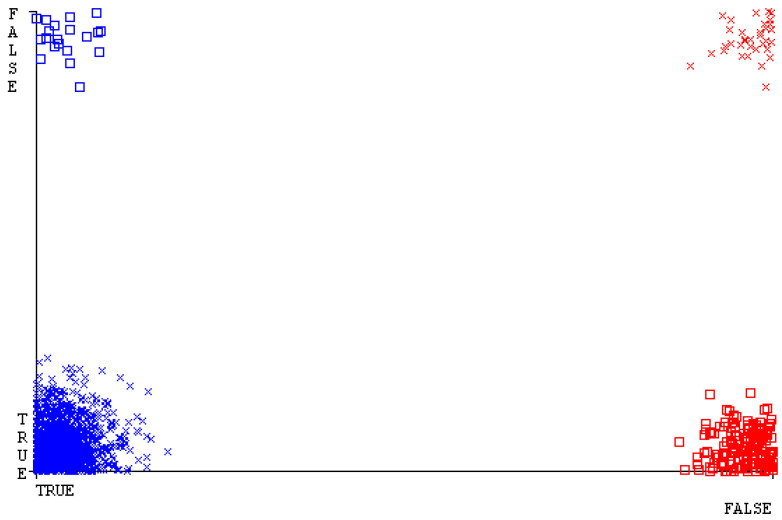
Classifier Errors of RF Classifier based on CPU-Mem Multi in Accuracy and Fault Prediction.

Figure 35, Figure 36, Figure 37, Figure 38, Figure 39 and Figure 40 represent the error of the classifier that shows the values corresponding to true-positive, true-negative, false-positive, and false-negative values. In Figure 35, Figure 36, Figure 37, Figure 38, Figure 39 and Figure 40, the square box represents the errors in the actual class versus the predicted class.

#### 5.2.3. Secondary Dataset HDD Mono Block-III

**Figure 41:**
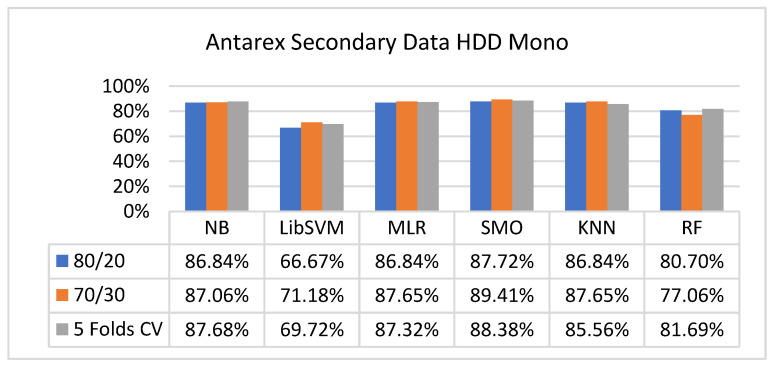
Accuracy by Class (True/False) of HDD Mono on ML Classifiers.

**Figure 42:**
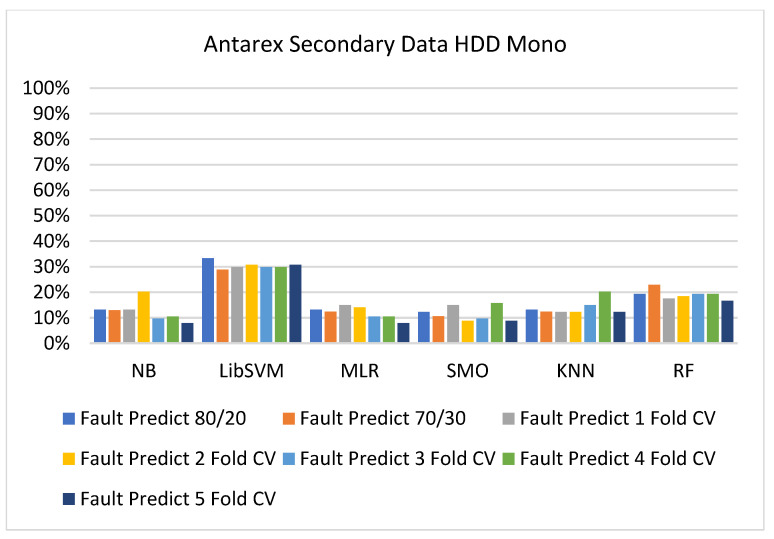
Fault Prediction by Class (True/False) of HDD Mono on ML Classifiers.

**Figure 43:**
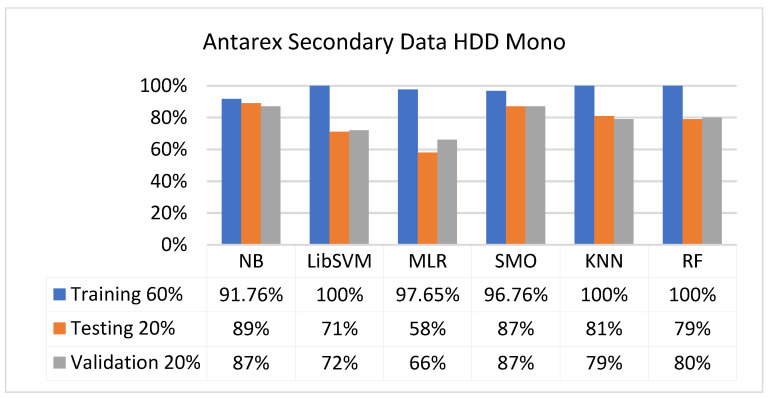
Accuracy by Class (True/False) of HDD Mono on ML Classifiers Related to Data Validation Results.

**Figure 44:**
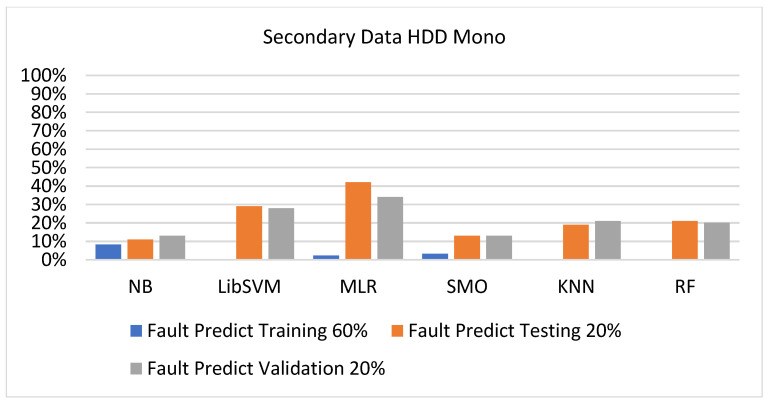
Fault Prediction by Class (True/False) of HDD Mono on ML Classifiers Related to Data Validation Results.

Figure 41, Figure 42, Figure 43 and Figure 44 depict a comparison of the results of NB, LibSVM, MLR, SMO, KNN, and RF in HDD mono related to detailed accuracy by class (true/false) and prediction on test-split additional data validation.

**Figure 45:**
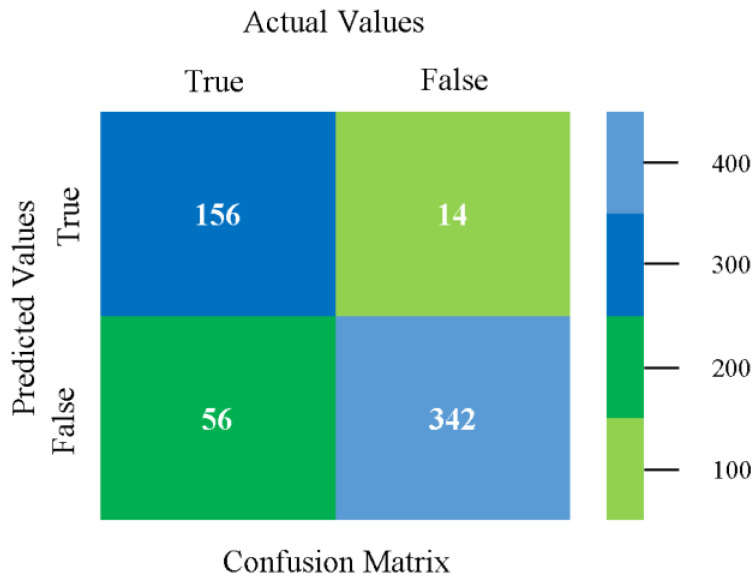
Confusion Matrix of NB Classifier based on HDD Mono in Accuracy and Fault Prediction.

**Figure 46:**
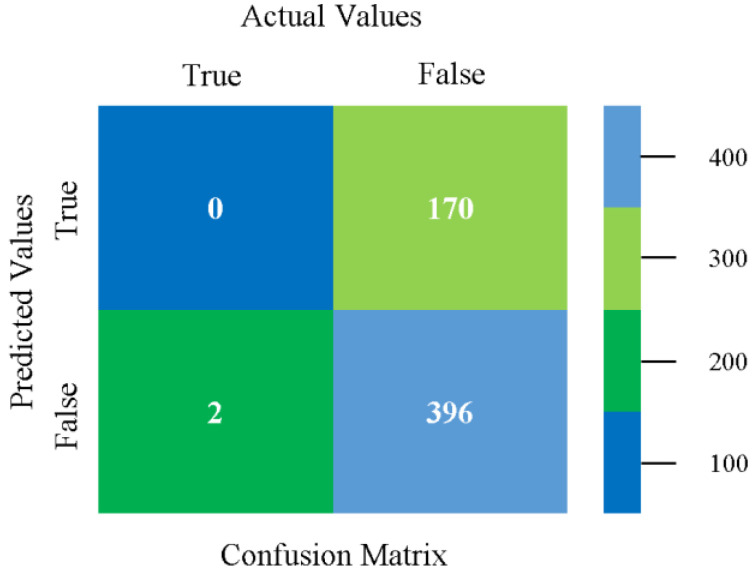
Confusion Matrix of LibSVM Classifier based on HDD Mono in Accuracy and Fault Prediction.

**Figure 47:**
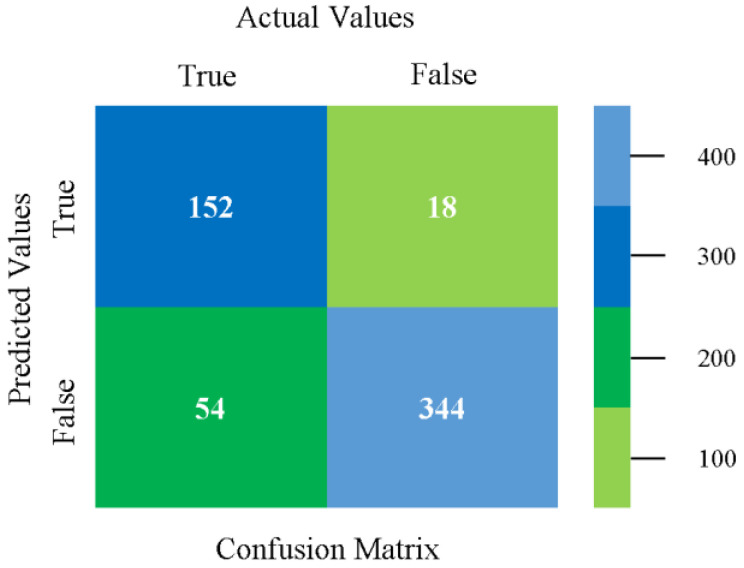
Confusion Matrix of MLR Classifier based on HDD Mono in Accuracy and Fault Prediction.

**Figure 48:**
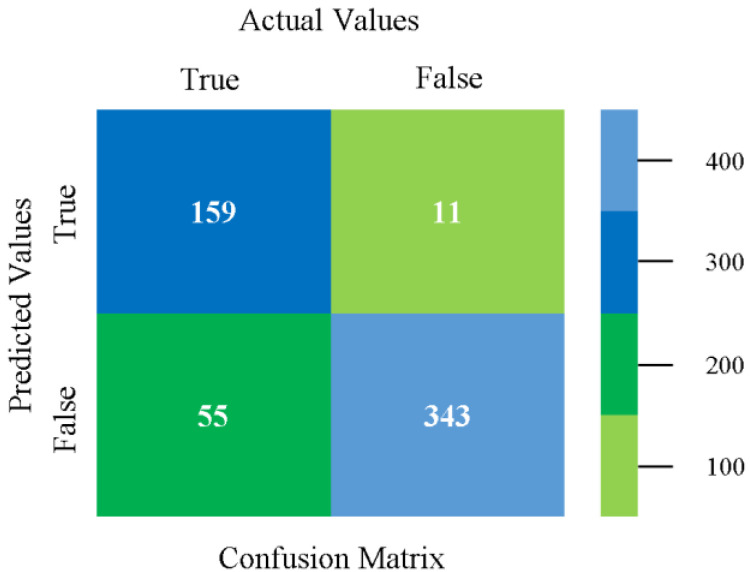
Confusion Matrix of SMO Classifier based on HDD Mono in Accuracy and Fault Prediction.

**Figure 49:**
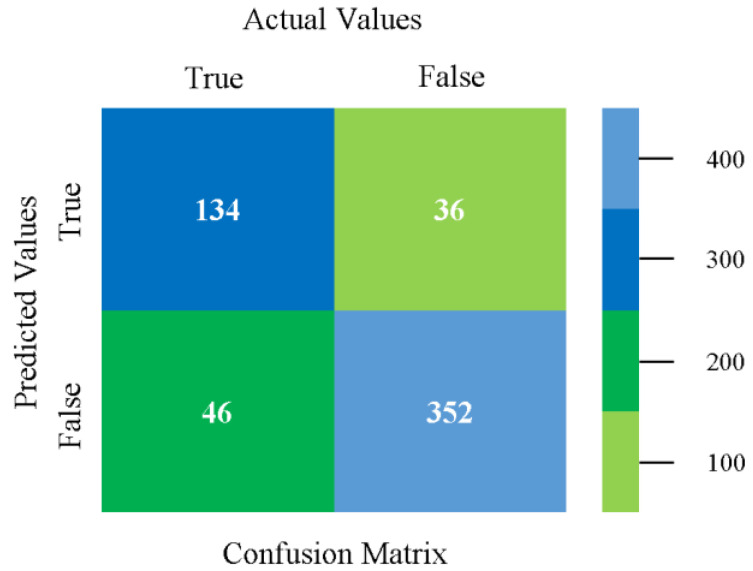
Confusion Matrix of KNN Classifier based on HDD Mono in Accuracy and Fault Prediction.

**Figure 50:**
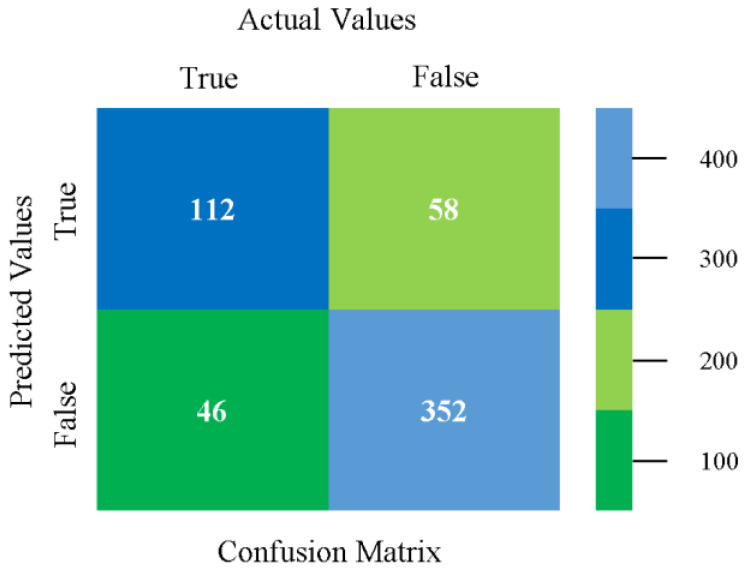
Confusion Matrix of RF Classifier based on HDD Mono in Accuracy and Fault Prediction.

The confusion matrix is used to calculate accuracy, precision, recall, and F-measure. It is used as an efficient technique for the classification of attributes based on qualitative response categories. Figure 45, Figure 46, Figure 47, Figure 48, Figure 49 and Figure 50 show the confusion matrix related to accuracy and fault prediction achieved through NB, LibSVM, MLR, SMO, KNN, and RF. The following confusion matrix indicates that the SMO classification model gave the highest percentage of accuracy and less fault prediction for HDD mono.

**Figure 51:**
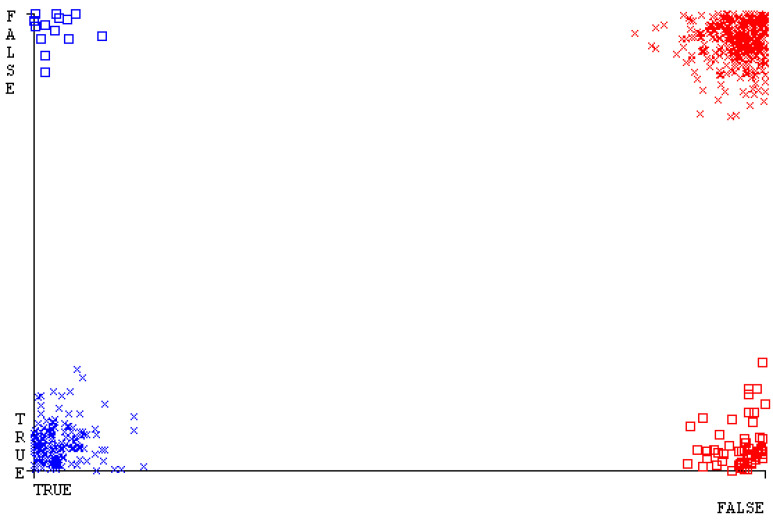
Classifier Errors of NB Classifier based on HDD Mono in Accuracy and Fault Prediction.

**Figure 52:**
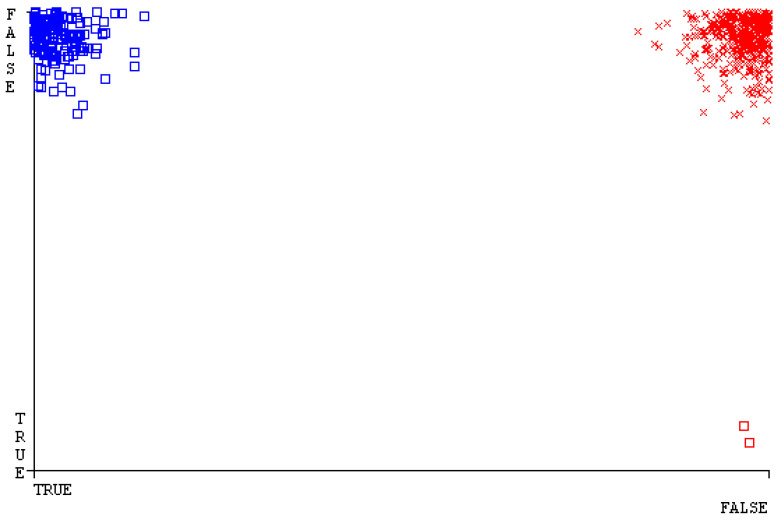
Classifier Errors of LibSVM Classifier based on HDD Mono in Accuracy and Fault Prediction.

**Figure 53:**
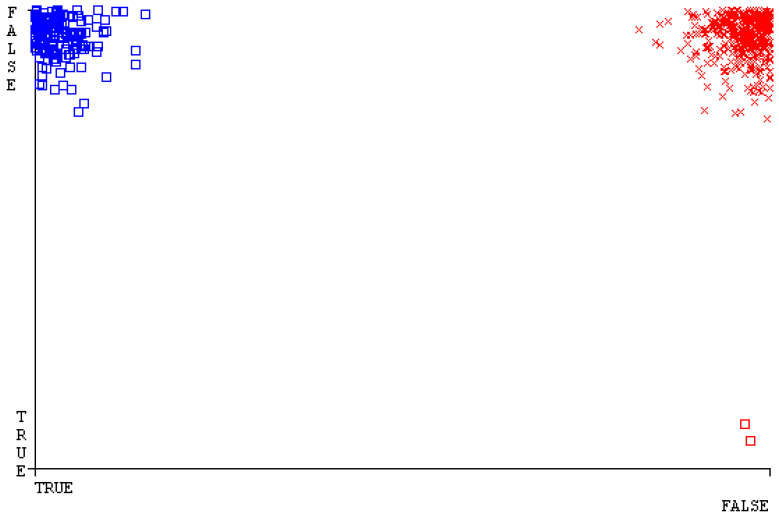
Classifier Errors of MLR Classifier based on HDD Mono in Accuracy and Fault Prediction.

**Figure 54:**
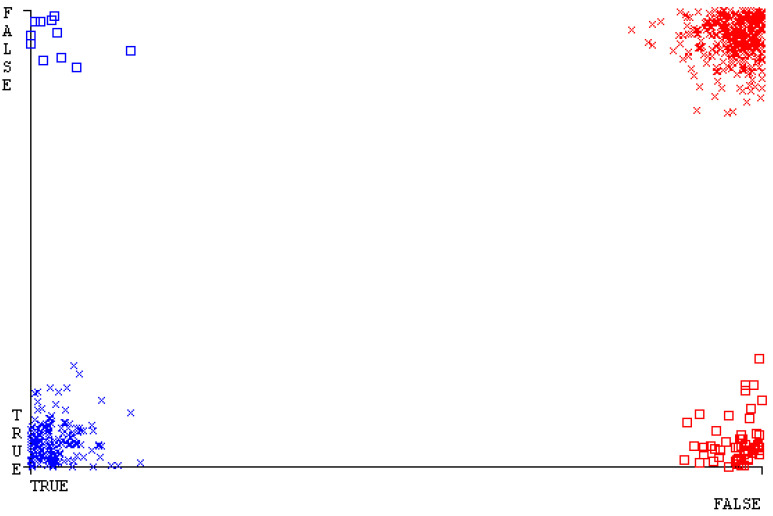
Classifier Errors of SMO Classifier based on HDD Mono in Accuracy and Fault Prediction.

**Figure 55:**
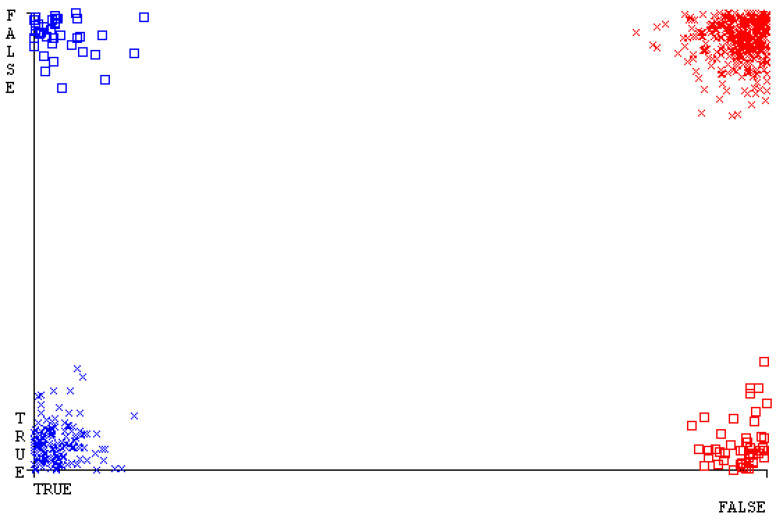
Classifier Errors of KNN Classifier based on HDD Mono in Accuracy and Fault Prediction.

**Figure 56:**
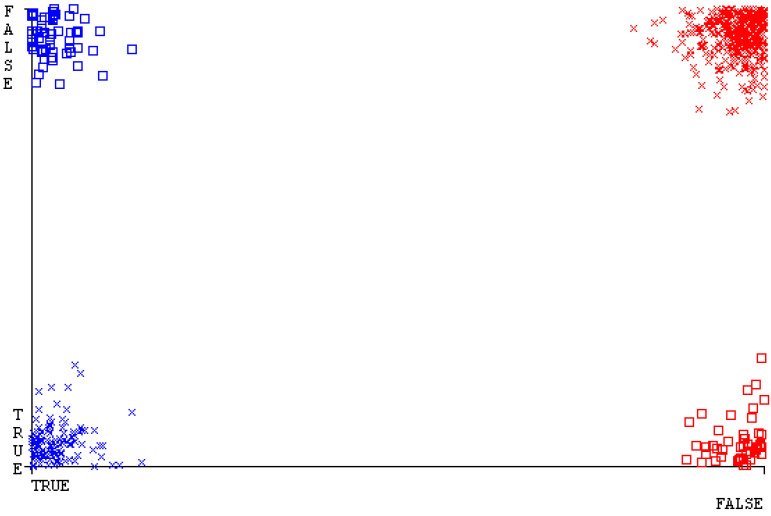
Classifier Errors of RF Classifier based on HDD Mono in Accuracy and Fault Prediction.

Figure 51, Figure 52, Figure 53, Figure 54, Figure 55 and Figure 56 represent the error of the classifier that shows the values corresponding to true-positive, true-negative, false-positive, and false-negative values. In Figure 51, Figure 52, Figure 53, Figure 54, Figure 55 and Figure 56, the square box represents the errors in the actual class versus the predicted class.

#### 5.2.4. Secondary Dataset HDD Multi Block-IV

**Figure 57:**
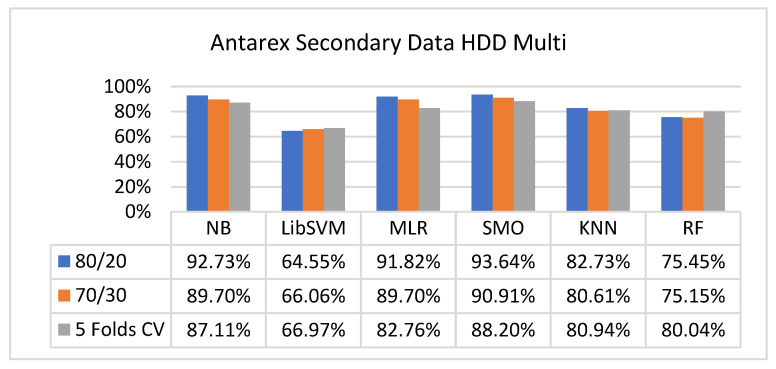
Accuracy by Class (True/False) of HDD Multi on ML Classifiers.

**Figure 58:**
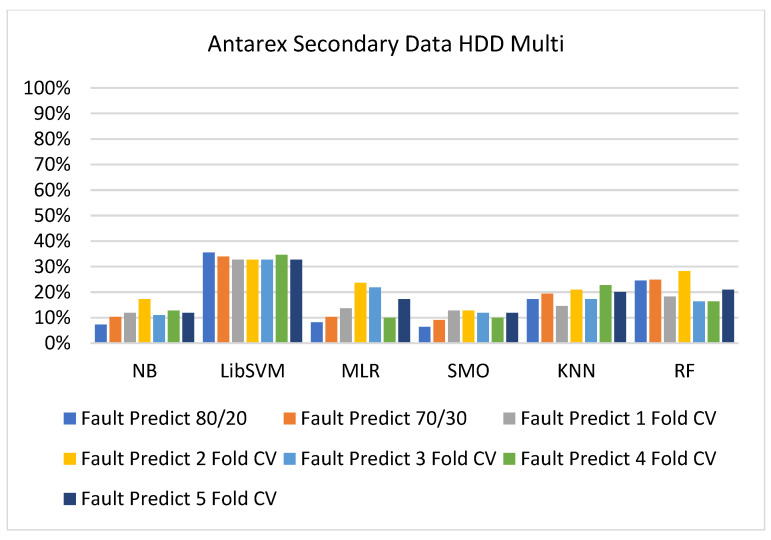
Fault Prediction by Class (True/False) of HDD Multi on ML Classifiers.

**Figure 59:**
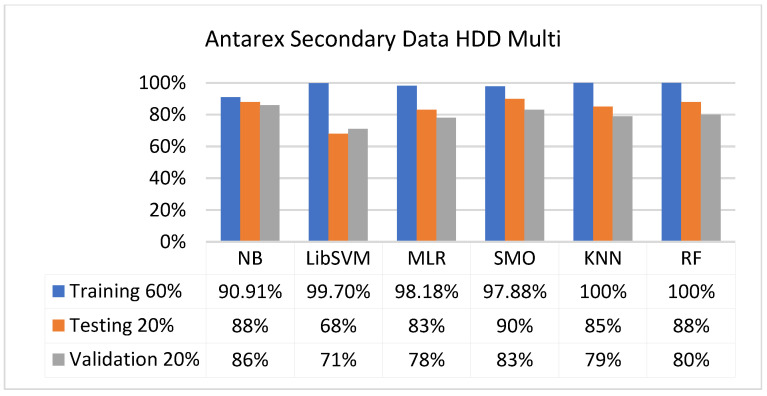
Accuracy by Class (True/False) of HDD Multi on ML Classifiers Related to Data Validation Results.

**Figure 60:**
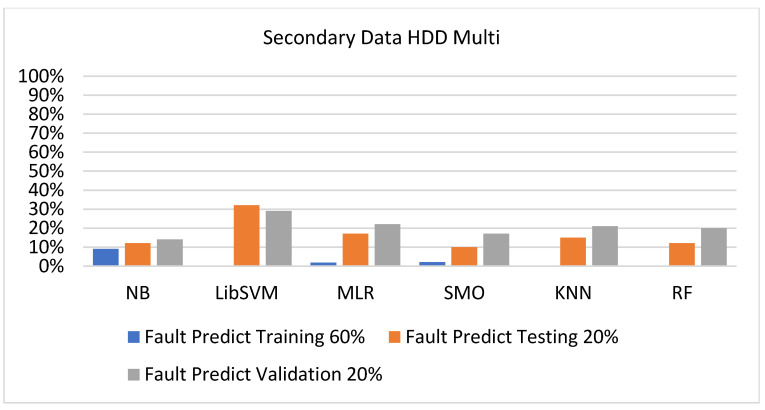
Fault Prediction by Class (True/False) of HDD Multi on ML Classifiers Related to Data Validation Results.

Figure 57, Figure 58, Figure 59 and Figure 60 show the result comparison of NB, LibSVM, MLR, SMO, KNN, and RF in HDD-multi-related detailed accuracy by class (true/false) and prediction on further test-split data validation.

**Figure 61:**
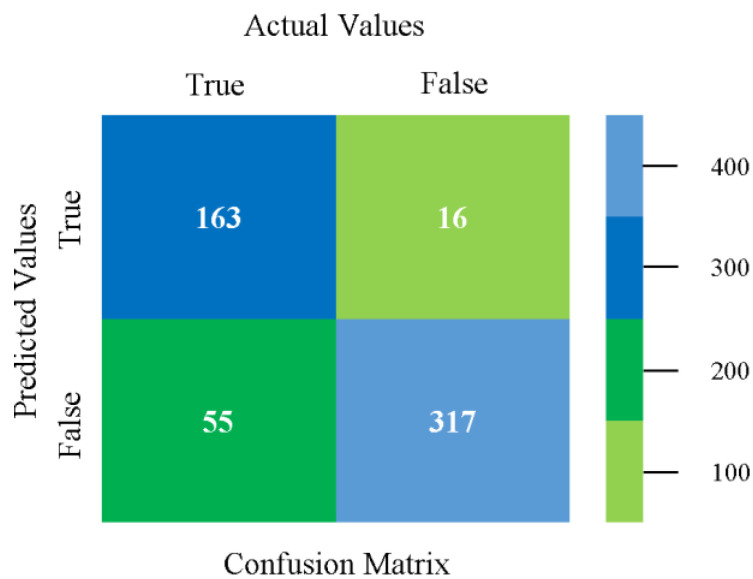
Confusion Matrix of NB Classifier based on HDD Multi in Accuracy and Fault Prediction.

**Figure 62:**
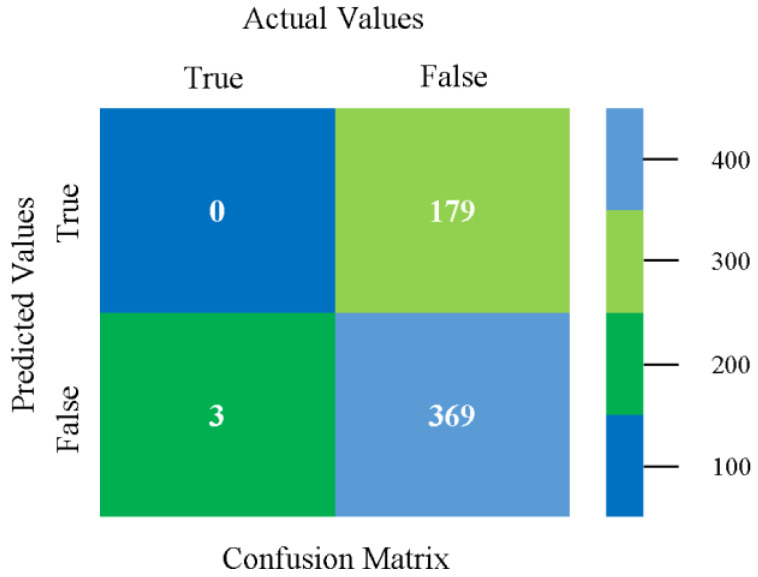
Confusion Matrix of LibSVM Classifier based on HDD Multi in Accuracy and Fault Prediction.

**Figure 63:**
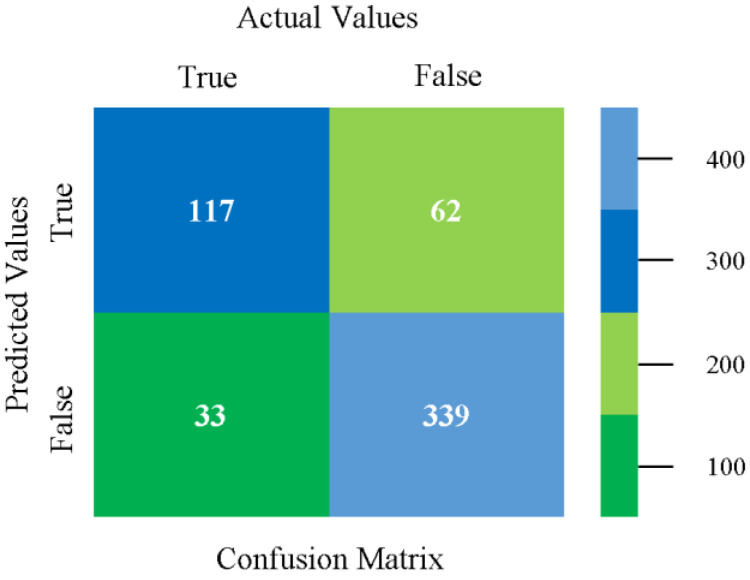
Confusion Matrix of MLR Classifier based on HDD Multi in Accuracy and Fault Prediction.

**Figure 64:**
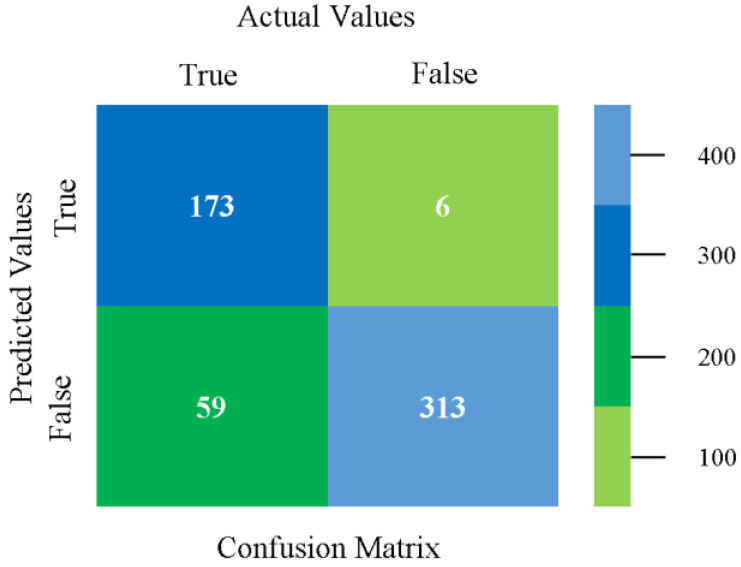
Confusion Matrix of SMO Classifier based on HDD Multi in Accuracy and Fault Prediction.

**Figure 65:**
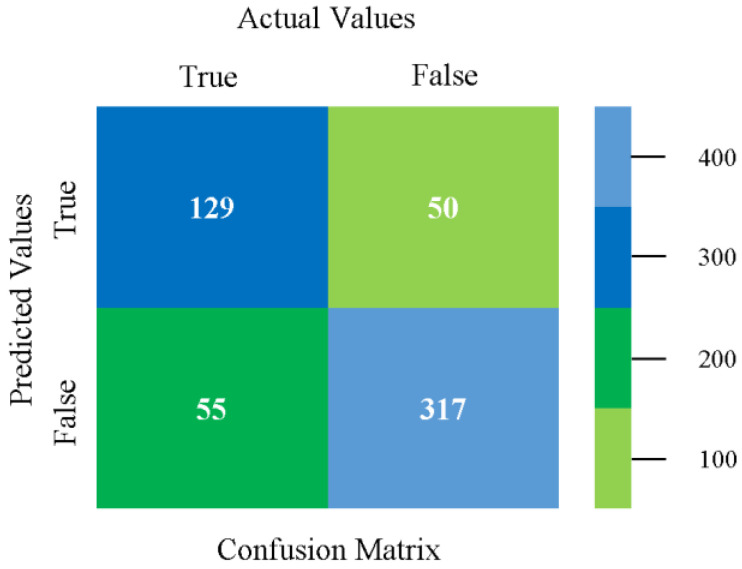
Confusion Matrix of KNN Classifier based on HDD Multi in Accuracy and Fault Prediction.

**Figure 66:**
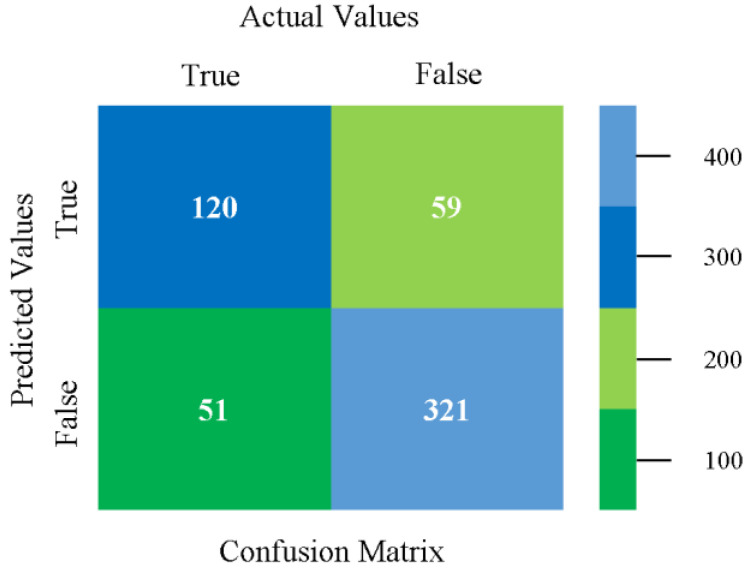
Confusion Matrix of RF Classifier based on HDD Multi in Accuracy and Fault Prediction.

The confusion matrix is used to calculate accuracy, precision, recall, and F-measure. It is used as an efficient technique for the classification of attributes based on qualitative response categories. Figure 61, Figure 62, Figure 63, Figure 64, Figure 65 and Figure 66 show the confusion matrix related to accuracy and fault prediction achieved through NB, LibSVM, MLR, SMO, KNN, and RF. The following confusion matrix indicates that the SMO classification model gave the highest percentage of accuracy and less fault prediction for HDD multi.

**Figure 67:**
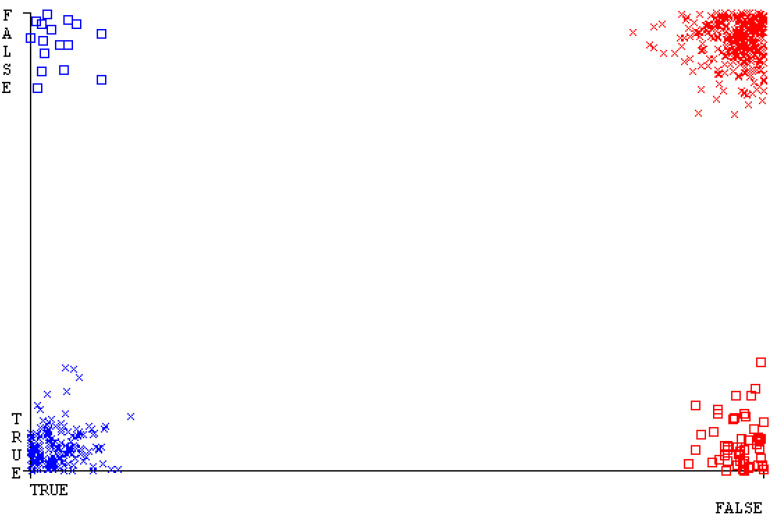
Classifier Errors of NB Classifier based on HDD Multi in Accuracy and Fault Prediction.

**Figure 68:**
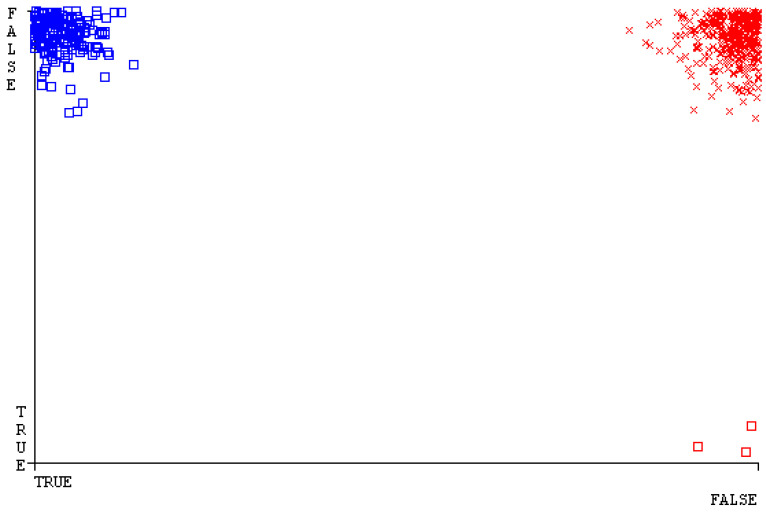
Classifier Errors of LibSVM Classifier based on HDD Multi in Accuracy and Fault Prediction.

**Figure 69:**
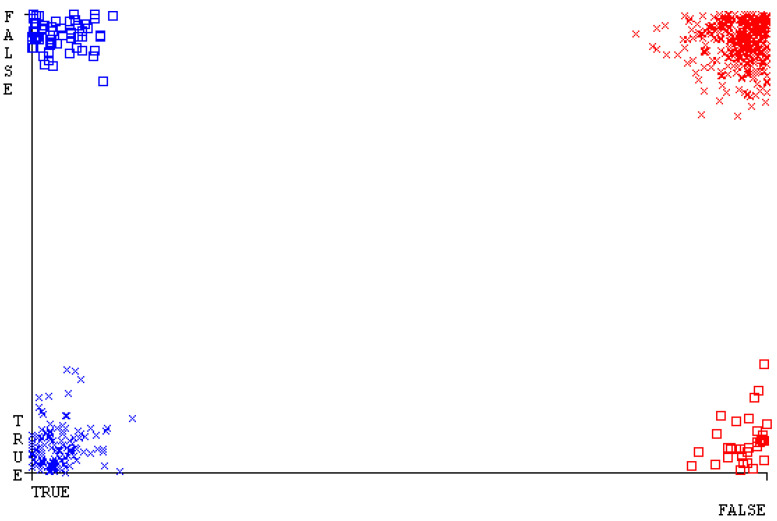
Classifier Errors of MLR Classifier based on HDD Multi in Accuracy and Fault Prediction.

**Figure 70:**
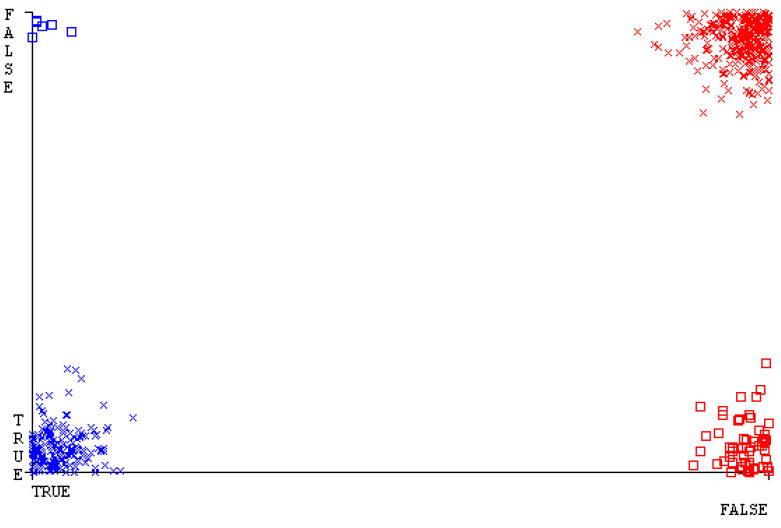
Classifier Errors of SMO Classifier based on HDD Multi in Accuracy and Fault Prediction.

**Figure 71:**
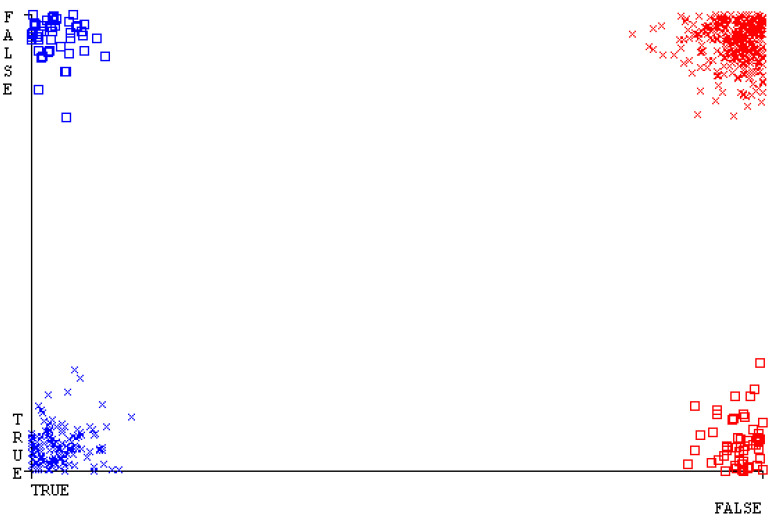
Classifier Errors of KNN Classifier based on HDD Multi in Accuracy and Fault Prediction.

**Figure 72:**
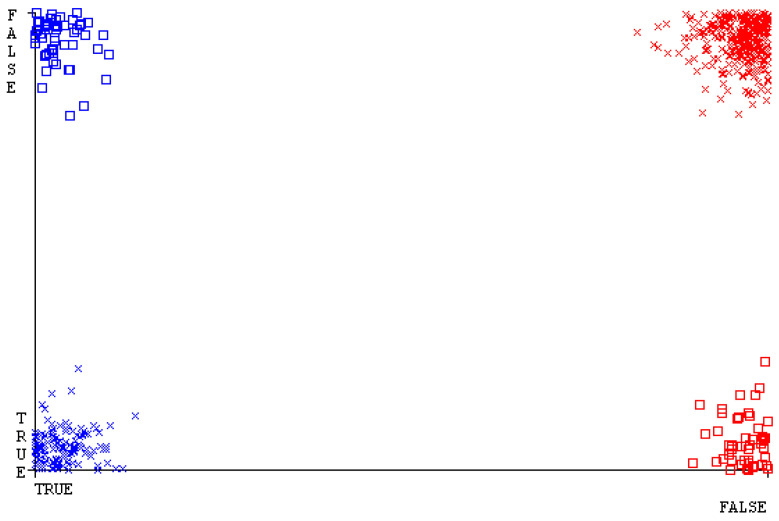
Classifier Errors of RF Classifier based on HDD Multi in Accuracy and Fault Prediction.

Figure 67, Figure 68, Figure 69, Figure 70, Figure 71 and Figure 72 represent the error of the classifier that shows the values corresponding to true-positive, true-negative, false-positive, and false-negative values. In Figure 67, Figure 68, Figure 69, Figure 70, Figure 71 and Figure 72, the square box represents the errors in the actual class versus the predicted class.

### 5.3. Comparison of Classification Models on Primary Dataset

We present the results associated with different classifiers using the STATUS class in the primary dataset. For classification models, we opted for NB, LibSVM, MLR, RF, KNN, and SMO with the poly kernel.

In the primary data results, we notice that the RF classifier gives the highest percentage of accuracy and less fault prediction in terms of 80/20 (97.14%), 70/30 (96.19%), and 5-fold cross-validation (95.85%), but the algorithm complexity (0.17 s) is not good. SMO gives the second highest accuracy and less fault prediction in terms of 80/20 (95.71%), 70/30 (95.71%), and 5-foldscross-validation (95.71%), and the algorithm complexity is good (0.3 s). The difference between the accuracy and lesser fault prediction between RF and SMO is just 0.13%, and the time complexity difference is 14 s.

**Figure 73:**
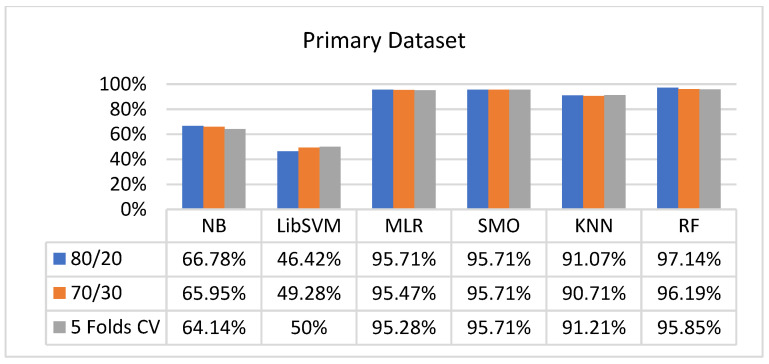
Accuracy by Class (Repair/Failure) of Primary Dataset on ML Classifiers.

**Figure 74:**
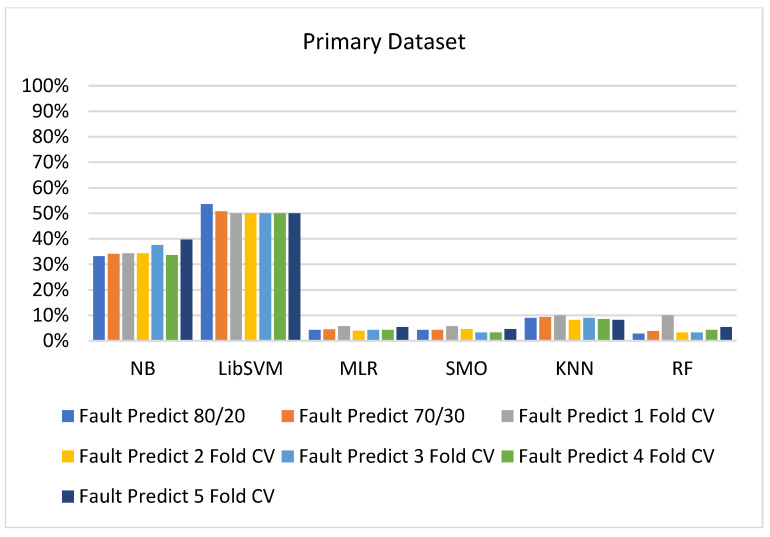
Fault Prediction by Class of (Repair/Failure) of Primary Dataset on ML Classifiers.

**Figure 75:**
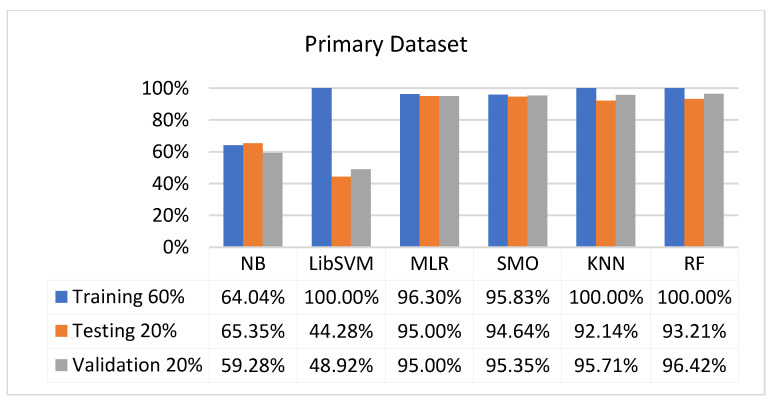
Accuracy by Class (Repair/Failure) of Primary Dataset on ML Classifiers Related to DV Results.

**Figure 76:**
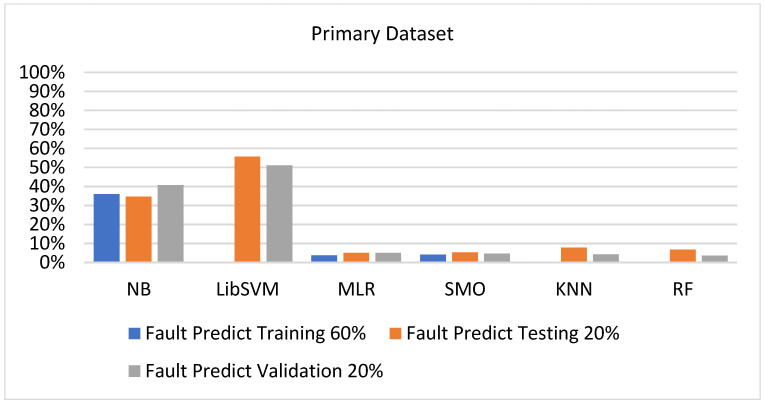
Fault Prediction by Class of (Repair/Failure) of Primary Dataset on ML Classifiers Related to DV Results.

Figure 73, Figure 74, Figure 75 and Figure 76 show the result comparison of NB, LibSVM, MLR, SMO, KNN, and RF in primary-dataset-related detailed accuracy by class (repair/failure) and prediction on further test-split data validation.

**Figure 77:**
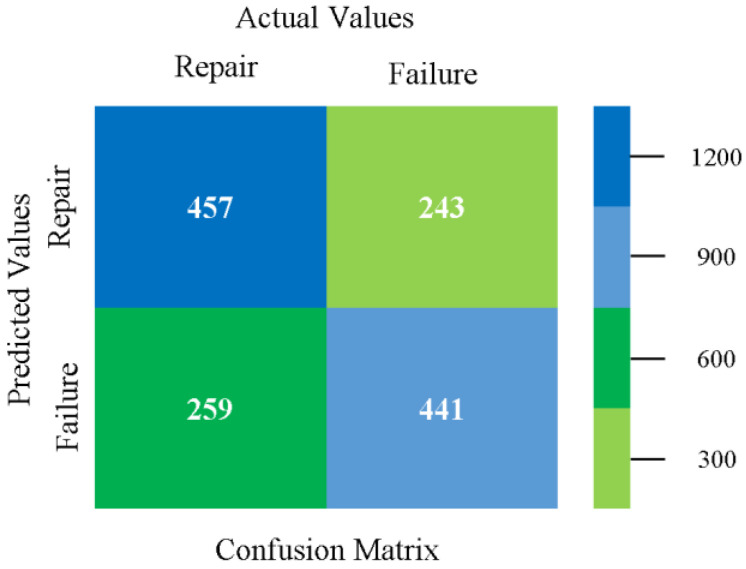
Confusion Matrix of NB Classifier based on Primary Data in Accuracy and Fault Prediction.

**Figure 78:**
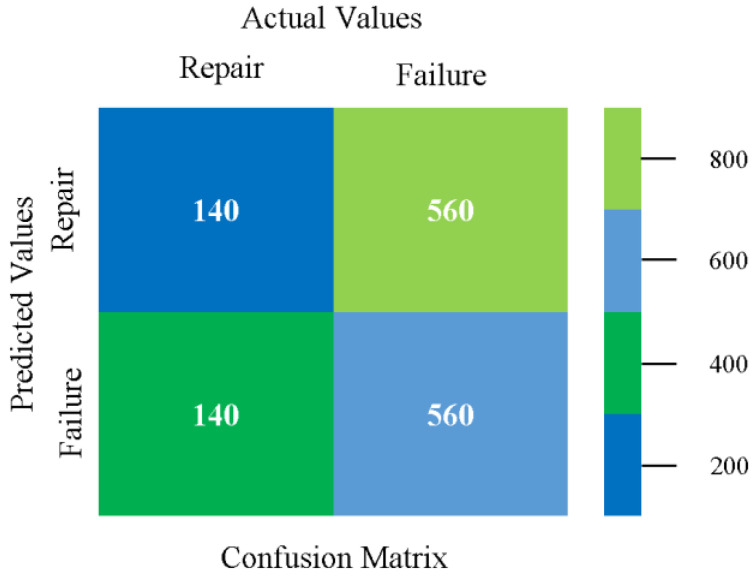
Confusion Matrix of LibSVM Classifier based on Primary Data in Accuracy and Fault Prediction.

**Figure 79:**
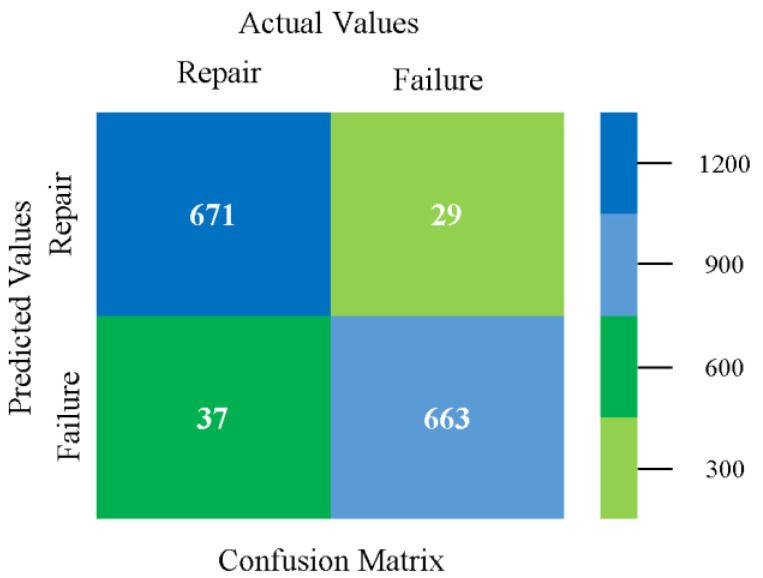
Confusion Matrix of MLR Classifier based on Primary Data in Accuracy and Fault Prediction.

**Figure 80:**
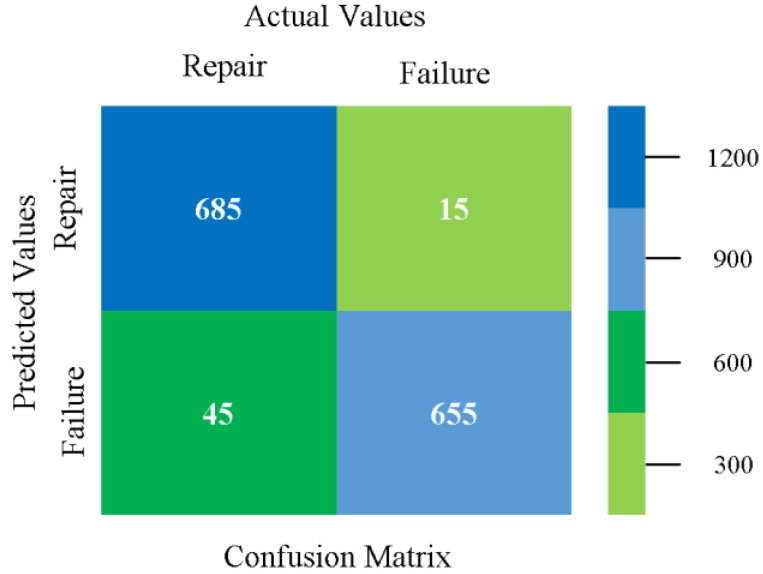
Confusion Matrix of SMO Classifier based on Primary Data in Accuracy and Fault Prediction.

**Figure 81:**
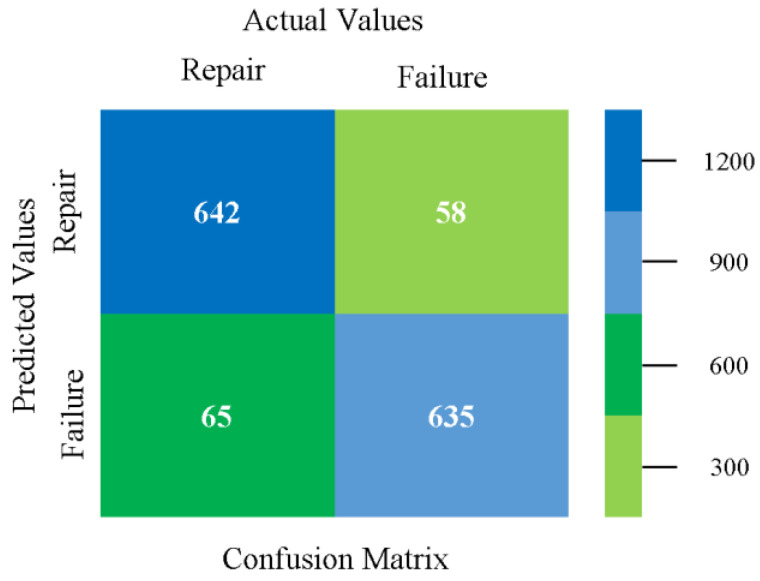
Confusion Matrix of KNN Classifier based on Primary Data in Accuracy and Fault Prediction.

**Figure 82:**
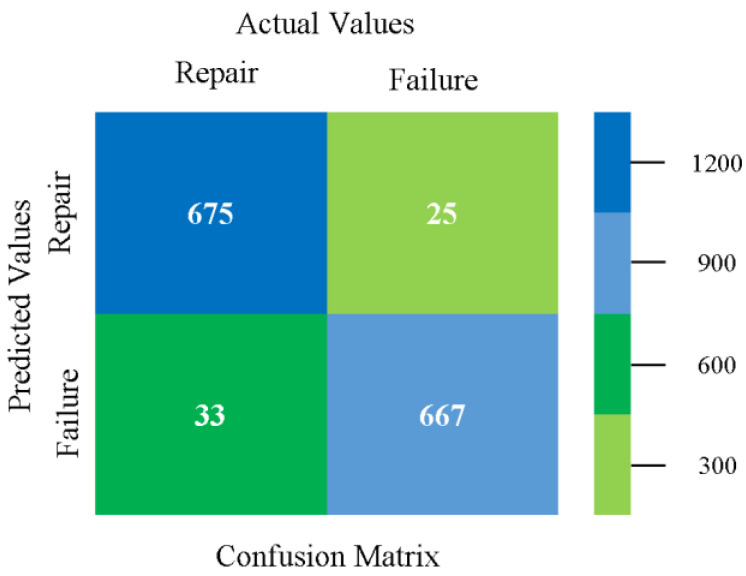
Confusion Matrix of RF Classifier based on Primary Data in Accuracy and Fault Prediction.

The confusion matrix is used to calculate accuracy, precision, recall, and F-measure. It is used as an efficient technique for the classification of attributes based on qualitative response categories. Figure 77, Figure 78, Figure 79, Figure 80, Figure 81 and Figure 82 show the confusion matrix related to accuracy and fault prediction achieved through NB, LibSVM, MLR, SMO, KNN, and RF. The following confusion matrix indicates that the RF classification model gave the highest percentage of accuracy and less fault prediction on the primary dataset, but the algorithm complexity (0.17 s) is not good.

**Figure 83:**
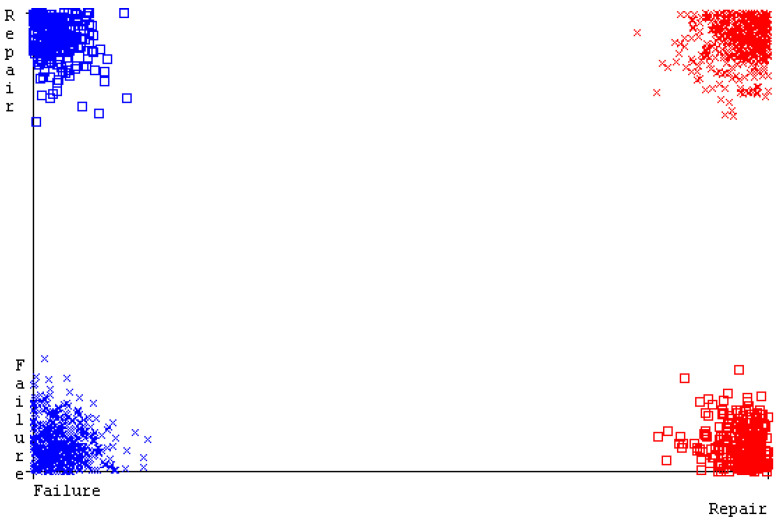
Classifier Errors of NB Classifier based on Primary Data in Accuracy and Fault Prediction.

**Figure 84:**
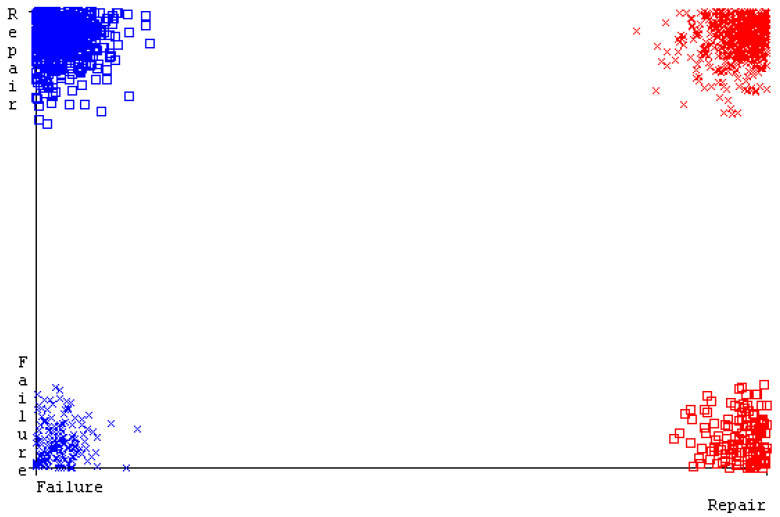
Classifier Errors of LibSVM Classifier based on Primary Data in Accuracy and Fault Prediction.

**Figure 85:**
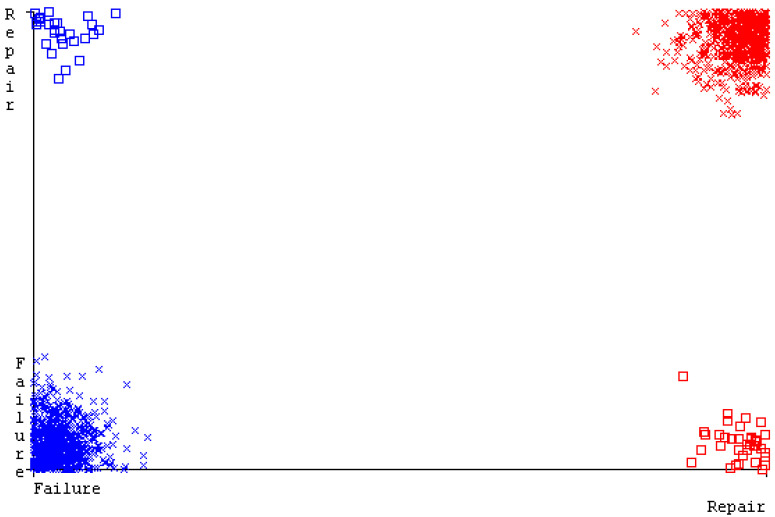
Classifier Errors of MLR Classifier based on Primary Data in Accuracy and Fault Prediction.

**Figure 86:**
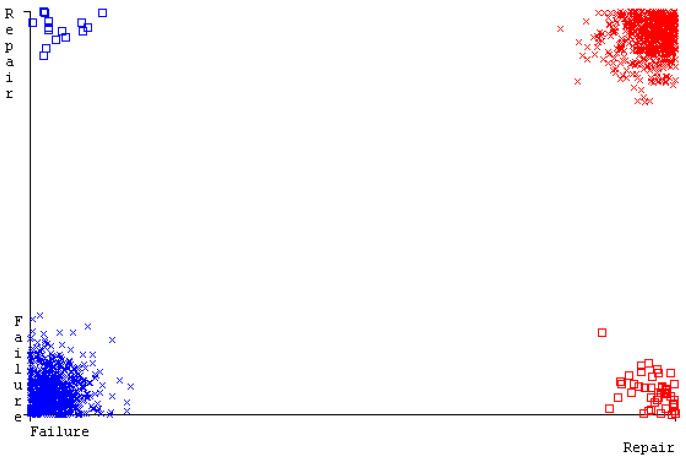
Classifier Errors of SMO Classifier based on Primary Data in Accuracy and Fault Prediction.

**Figure 87:**
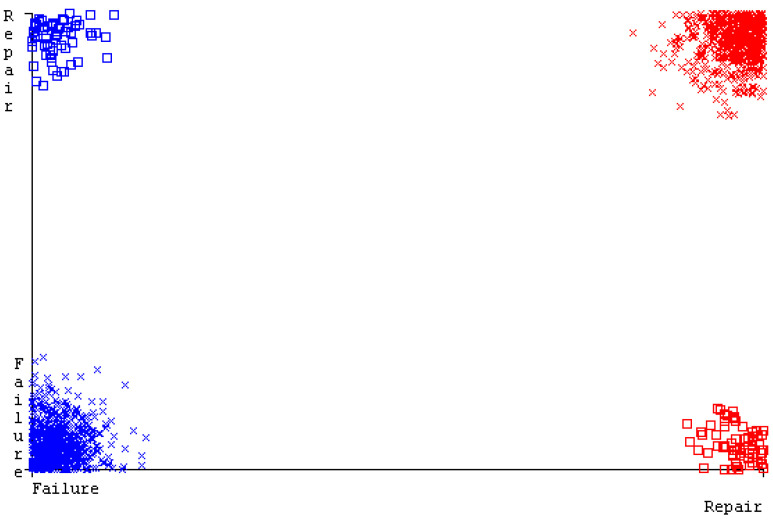
Classifier Errors of KNN Classifier based on Primary Data in Accuracy and Fault Prediction.

**Figure 88:**
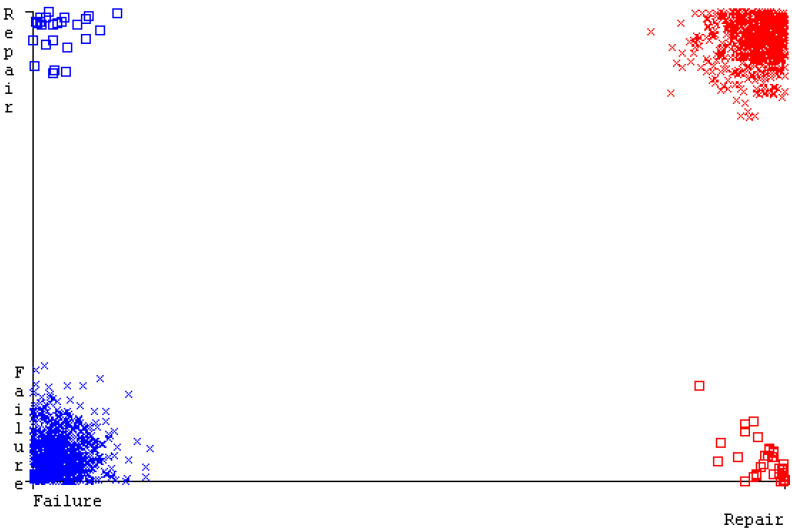
Classifier Errors of RF Classifier based on Primary Data in Accuracy and Fault Prediction.

SMO gives the second-highest accuracy and less fault prediction, and the algorithm complexity is good (0.3 s). The difference in accuracy and lesser fault prediction between RF and SMO are just 0.13%, and the time complexity difference is 14 s. Figure 83, Figure 84, Figure 85, Figure 86, Figure 87 and Figure 88 represent the error of the classifier that shows the values corresponding to true-positive, true-negative, false-positive, and false-negative values. In Figure 83, Figure 84, Figure 85, Figure 86, Figure 87 and Figure 88, the square box represents the errors in the actual class versus the predicted class.

### 5.4. Modified Sequential Minimal Optimization Results

**Figure 89:**
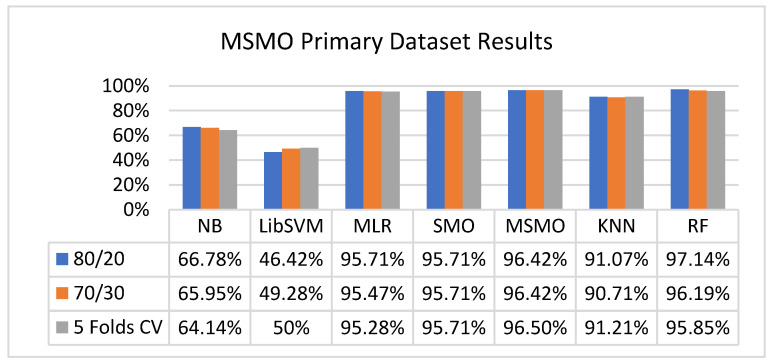
Comparison of ML Classifiers with MSMO Accuracy by Class (Repair/Failure) of Primary Dataset.

**Figure 90:**
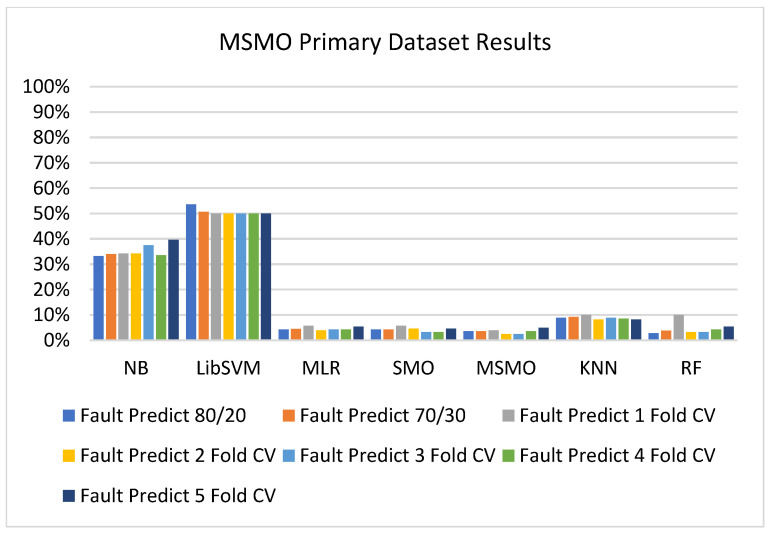
Comparison of ML Classifiers with MSMO Fault Prediction by Class (Repair/Failure) of Primary Dataset.

**Figure 91:**
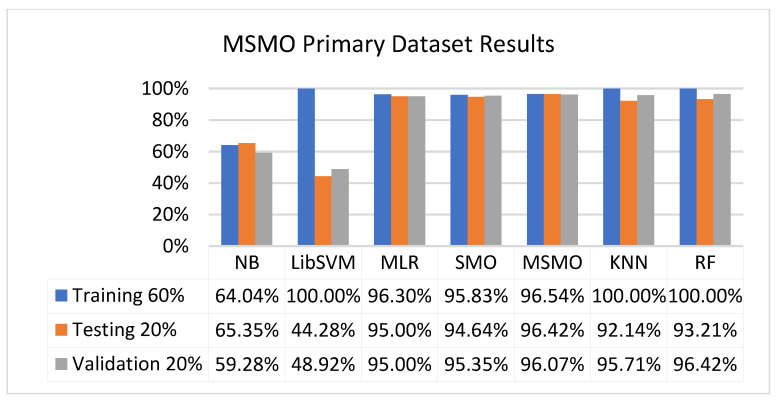
Comparison of ML Classifiers with MSMO Accuracy by Class of Primary Dataset Related to DV Results.

**Figure 92:**
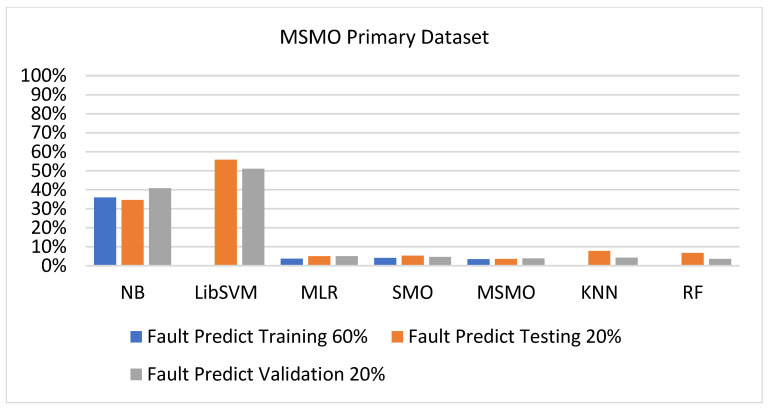
Comparison of ML Classifiers with MSMO Fault Prediction by Class of Primary Dataset Related to DV Results.

In this subsection, the results of the classification of the primary dataset results are shown in Figure 89, Figure 90, Figure 91 and Figure 92, indicating that the MSMO classification model gives the highest accuracy and less fault prediction in terms of 80/20 (96.42%), 70/30 (96.42%), and 5-fold cross-validation (96.50%). The MSMO time complexity of the algorithm is 0.44 s after modification.

Figure 89, Figure 90, Figure 91 and Figure 92 show the result comparison of NB, LibSVM, MLR, SMO, MSMO, KNN, and RF in primary-dataset-related detailed accuracy by class (repair/failure) and prediction on further test-split data validation.

**Figure 93:**
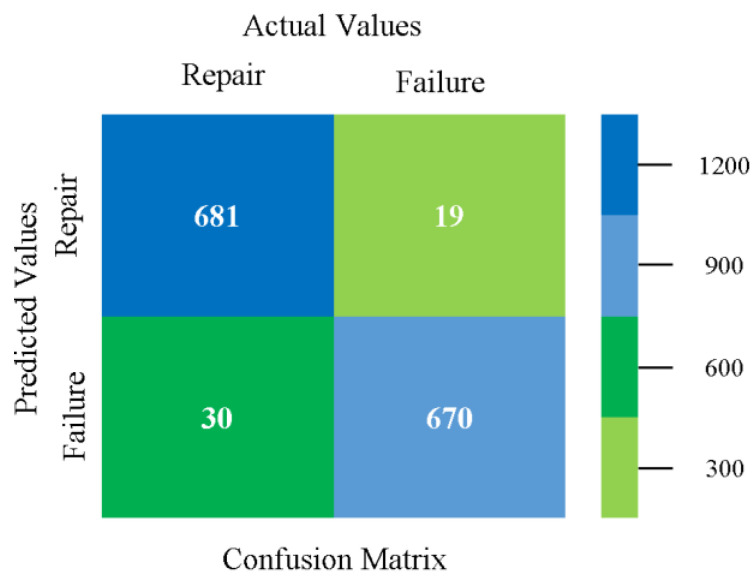
Confusion Matrix of MSMO Classifier based on Primary Data in High Accuracy and Less Fault Prediction Error.

The confusion matrix is used to calculate accuracy, precision, recall, and F-measure. It is used as an efficient technique for the classification of attributes based on qualitative response categories. Figure 93 shows the confusion matrix related to accuracy and fault prediction achieved through MSMO. The following confusion matrix indicates that the MSMO classification model gave the highest percentage of accuracy and less fault prediction error for the primary dataset against NB, LibSVM, MLR, SMO, KNN, and RF.

**Figure 94:**
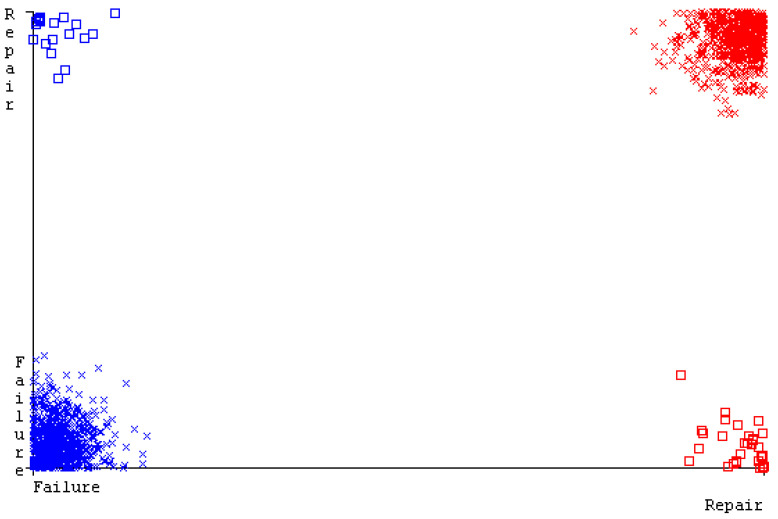
Classifier Errors of MSMO Classifier based on Primary Data in Accuracy and Fault Prediction.

Figure 94 represents the error of the classifier that shows the values corresponding to true-positive, true-negative, false-positive, and false-negative values. In the Figure 94, the square box represents the errors in the actual class versus the predicted class.

### 5.5. Simulation Setup of D-CP to Achieve Reliability

To achieve reliability, we integrated the MSMO classifier results with the D-CP fault-tolerance technique. D-CP can learn from previous data and execute them.

We used a cloud simulation 3.0.3 toolkit. It is a simulation tool to mimic CC scenarios. We extended the cloud simulation simulator with a fault-tolerance D-CP method to achieve reliability. Table 7 shows the hardware specifications for an experiment.

### 5.6. Delta-Checkpointing Results

In this step, we configured the CP configuration file, the number of cloud users, and the cloud simulation library; created a data center, a broker, and a cloudlet; and submitted the VM list to the broker using the D-CP method. A data center with a recovery scheduler, CP scheduler, CP image index, data center destroyer, VMs, and cloudlets was included in the simulated platform. Each simulated VM has unique properties in our execution environment. Table 8 shows the parameters that affect D-CP reliability through VMs.

The results of D-CP techniques for achieving reliability are shown in Table 9. It indicates that all VMs were successfully executed without VM failure because the D-CP mechanism regularly saves the VMs execution state as a CP image during failure-free execution. In the event of a failed event, the VM is restarted from an intermediate state using the previously saved CP image. The amount of computation lost as a result is reduced. The status (success/failure) determines the dependability of the multiple nodes VMs. Multiple nodes, all of which are reliable VMs, were successfully executed. Table 9 shows the results of achieved reliability through the ML & D-CP approach. 

## 6. Discussion

It is not easy to achieve the FT model’s reliability in CC. Only a few FT models are based on reliability. Furthermore, ML has not been extensively considered for FT reliability. There was a need to design and implement a model using ML and FT methods that achieved high accuracy, less fault prediction, and maximum reliability.

This study was conducted to achieve high accuracy, less fault prediction, and achieve reliability. To ensure the smooth execution of the research, the MSMO classifier with a D-CP approach was developed.

The primary data were subjected to an MSMO classifier. MSMO classifier results show that the proposed strategy outperformed the existing classifier in terms of accuracy and fault prediction. In the primary data, the obtained results were compared to the existing NB, LibSVM, MLR, SMO, KNN, and RF classifiers. The most important parameter for judging the classifier’s performance level is high accuracy with less fault prediction. To eliminate VM failure, the MSMO classifier results were integrated into the delta-checking approach.

Simulated results were compared with NB, LibSVM, MLR, SMO, KNN, and RF classifiers, and it was proven that the proposed classifier performed more accurately and rapidly, with 96.5% correctly classified instances compared to the available classifiers. The innovation of the proposed research is in the many techniques that were correlated to high accuracy and less fault prediction to achieve reliability. MSMO was proposed by applying parameter tuning, which can be acknowledged as a novel approach, and D-CP was proposed by applying different parameters of CP.

## 7. Conclusions

The data was analyzed and the results were presented in section five. Section five includes classification results from NB, LibSVM, MLR, SMO, and RF with a confusion matrix and graphical representations. Finally, the MSMO results, which are the main algorithm of this research study, are included here. This research compares traditional, ML, and FT techniques in terms of high accuracy, low fault prediction error, and reliability.

The secondary dataset was collected from the homonymous experimental HPC system at ETH Zurich during fault injection and is used by researchers to conduct ML-based fault prediction studies. The dataset is divided into two sections: one for CPU and memory benchmark applications and fault programs, and another for hard drive-related applications and fault programs. The dataset archive is divided into four directories, one for each dataset block (CPU/Memory and HDD in single-core and multi-core variants). According to the findings, there is a significant difference in the four directories of the secondary dataset. The distinction is that CPU-Mem Multi-cores outperform the other directories.

The primary dataset was created using the Weibull distribution method. The Weibull distribution is a popular time-to-failure model for reliability. It adds non-constant failure rate functions to the exponential model. The model, which includes both rising and falling failure rate curves, has been used to successfully explain both initial burn-out and wear-out failures. Based on the comparisons, the primary dataset outperforms the secondary dataset, implying that the primary dataset is adequate for modifying the ML algorithm in this study. This study demonstrated that the ML-based approach significantly improved FT reliability in CC, resulting in higher accuracy and less fault prediction for users. The pr posed approach increases the credibility of the FT for reliability.

### 7.1. Research Contribution

This research study made the following significant contributions:

1. The challenges of CC and challenges of FT that may compromise the success of reliability in the CC environment were identified from the literature review;
2. The reliability of VMs in terms of node failure could have a negative impact on users;
3. The MSMO classifier and D-CP FT approach was used to achieve high accuracy, less fault prediction, and successful execution of VMs, which all have a positive impact on users in the CC environment.

### 7.2. Limitations

1. Antarex secondary data collection is possible, but more computational resources are required because this is an HPC fault dataset; however, we can download this dataset through the ZONODO website;
2. The Weibull distribution was not provided to generate a fault dataset for primary data generation;
3. An effort was made to achieve the primary dataset using the Weibull distribution;
4. Achievement of high accuracy and less fault prediction compared to the proposed MSMO classifier results was not available in the computing environment to prove the reliability of the results.

### 7.3. Future Directions

1. Using the Weibull distribution approach, a graphical user interface can be created to generate the primary dataset in cloud simulation 3.0.3;
2. Tuning parameters can be automatically adjusted using code, but keep in mind that to find the best tuning parameter value, the code must not become stuck;
3. Random forest can be implemented to achieve high accuracy and low fault prediction, but more work on the algorithm’s complexity is required. Comparative analysis can also be performed with this proposed work;
4. Deep learning algorithms can also be used to achieve high accuracy while predicting fewer faults. The sample size should be increased. The larger the sample size, the more accurate and reliable the results. When the dataset is large, DL techniques outperform ML techniques;
5. Reinforcement learning can be used to implement or improve the FT capability of a system. Such ideas are easily adaptable to cloud environments;
6. To achieve reliability, the D-CP approach can be combined with deep learning and reinforcement learning.

## Figures and Tables

**Table 1:** A Summary of the Literature Review.

Ref	Author Name	Year	Benefits	Drawbacks
[12]	Muhammad Asim Shahid et al.	2020	They identify the need for FT efficiency metrics in algorithms in this article, which is one of the main concerns in cloud environments.	They do not provide quality of service in terms of reliability.
[13]	Vipul Gupta et al.	2019	In this article, they show that the accuracy value of the fault tolerance is 79%, which is better than in the existing method.	They do not provide classification techniques for selecting fault-tolerance nodes based on virtual machine success/failure.
[14]	Rakesh et al.	2020	In this article, reactive FT mechanisms were found to likely result in failure.	In this article, they do not implement machine learning algorithms for better fault prediction, so they are not providing high accuracy and less fault prediction.
[15]	Sam Goundar and Akashdeep Bhardwaj	2018	This article discusses fault-tolerance systems for cloud computing environments and examines whether or not they are effective in a cloud environment.	They do not address accuracy and fault prediction to achieve reliability.
[16]	Mihiretu Kebede Edemo	2019	The author created a fault-tolerance architecture that can effectively use versions in real-time cloud computing systems.	The limitation is that the architecture cannot tolerate faults if an equal number of versions fail in each subpart at the same time, especially if the number of failed versions exceeds the number of error-free versions in all subparts.
[17]	Jackson Kamiri and Geoffrey Mariga	2021	The primary goal of this paper was to investigate current machine learning research methods, emerging themes, and the implications of those themes in machine learning research.	They do not offer content analysis for machine learning applications such as supervised learning, text analytics, classification, and prediction.
[18]	Iqbal H. Sarker	2021	The author provides a comprehensive overview of machine learning algorithms, which can be used to improve an application’s intelligence and capabilities.	There is a lack of analysis on machine learning algorithms.
[19]	Umer Ahmed Butt et al.	2020	They present an analysis of CC security threats, issues, and solutions that used one or more ML algorithms in this review paper.	There is a lack of a proposed solution to achieve reliability based on VM failure.
[20]	Shiliang Sun et al.	2019	In this article, they use of ML algorithms to improve accuracy.	There is a lack of challenges and open problems in ML optimization methods.
[21]	Deepak Kochhar et al.	2017	The proactive fault-tolerance technique is used in this article, and they propose using the NB classifier to classify the nodes.	There is a lack of use of other classification algorithms to improve accuracy and achieve less fault prediction.
[22]	Chih-Chung Chang and Chih-Jen Lin	2022	In this article, they present the implementation of LibSVM and discuss all issues.	There is a lack of ensuring good system reliability.
[23]	Nor Amira Mohamad et al.	2016	This study used MLR model to determine fault prediction.	There is a lack of use of other classification algorithms to determine fault prediction.
[24]	C.R. LI and J. GUO	2015	The authors of this paper proposed an improved version of SVM that can avoid falling into endless loops.	The article was unable to determine the optimal parameter in an n-way that can speed up training.
[25]	Pratap Chandra Sen et al.	2020	This paper attempts to compare various types of classification algorithms and provides a thorough review of all supervised learning classifications.	There is a lack of a proposed solution to achieve reliability based on VM failure.
[26]	Salma M.A. Attallah et al.	2020	The main goal of the proposed model is to track changes in CPU utilization and make a decision when a high value of CPU utilization is identified.	There is a lack of a proposed solution to achieve reliability based on VM failure.
[27]	S. Suguna and K. Devi	2015	The authors proposed a virtual machine fault-tolerance technique in this article to achieve reliability.	In this article, the authors only achieved one virtual machine result that was successful, and the remaining were failures.

**Table 2:** Short Overview of the Secondary Dataset.

Dataset Directories	Attributes	Attributes Names + Types				Instances
CPU-Mem Mono	8	1. Timestamp	Nume	2. Type	Nomi	4005
CPU- Mem Multi	8	3. Args	Nomi	4. Seqnum	Nume	4380
HDD Mono	8	5. Duration	Nume	6. Cores	Nume	3244
HDD Multi	8	7. Error	Nomi	8. isFault	Nomi	2493

**Table 3:** Overview of the Parameters of Primary Data Generated.

User	Port NO	Host NO	Network Host	Distribution
1	16	192	Mips, Ram, Storage, and Bandwidth	Weibull (this includes both rising and decreasing failure rate functions).

**Table 4:** Short Overview of the Primary Dataset.

Attributes	Attributes Names		Attributes Type	Instances
7	9. FHID	10. HFTIME	Numeric and Nominal	1400
	11. LFT	12. DIS		
	13. DISHT	14. FTIME/RTIME		
	15. STATUS			

**Table 5:** Parameter Configuration of ML Classifiers.

Classifiers	Configuration Parameters	Values
NB	Batch size	100
	Debug	False
	Display model in old format	False
	Do not check capabilities	False
	Num decimal places	2
	Use kernel estimator	False
	Use supervised discretization	False
LIBSVM	SVM type	C-SVC (Classification)
	Degree	3
	EPS	0.001
	Gamma	0.0
	Kernel type	radial basic function
	Normalize	False
	Seed	1
MLR	Batch size	100
	Do not check capabilities	False
	Num decimal places	4
	Ridge	1.0 × 10^−8^
SMO	C complexity parameter	1.0
	Epsilon	1.0 × 10^−12^
	Filter type	normalize training data
	Kernel	Polykernel −10 1.0−C 25,007
	Num folds	1
	Random seed	1
	Tolerance parameter	0.001
KNN	KNN	1
	Batch size	100
	Cross validate	False
	Nearest neighbor search algorithm	linear NN search
RF	Batch size	100
	Max depth	0
	Num decimal places	2
	Num features	0
	Num iterations	100
	Seed	1

**Table 6:** Configuration Description of Experiment.

System	
Processor	Intel (R) Core (TM) i7 2620M CPU 2.7 GHz
RAM	8.00 GB
Windows	8.1 Platform
IDE	WEKA (3.8.6)
Weka Interface	Explorer
Java Version	17.0.2

**Table 7:** Hardware Specifications for Experiment.

System	
Processor	Intel (R) Core (TM) i7 2620M CPU 2.7 GHz
RAM	8.00 GB
Windows	8.1 Platform
IDE-1	Eclipse IDE for Java Developer Release 2021-09 (4.21.0)
IDE-2	Cloud simulation 3.0.3

**Table 8:** Parameters of D-CP and VMs.

Parameters	Values
Number of cloud users	1
Number of data centers	1
Number of VMs	11
VM frequency	1000 MIPS
VM memory (RAM)	4 GB
VM bandwidth	10 Gbps
VM storage	1000 GB

**Table 9:** Shows Achieved Reliability Results Based on ML and D-CP.

Cloudlet ID	Status	DC ID	VM ID	Time	Start Time	Finish Time
69	SUCCESS	1029	282	36,534	0	36,534
98	SUCCESS	1029	1385	36,640	0	36,640
74	SUCCESS	1029	780	37,189	0	37,189
40	SUCCESS	1029	1121	37,575	0	37,575
105	SUCCESS	1029	774	37,855	0	37,855
0	SUCCESS	1029	2992	38,030	0	38,030
44	SUCCESS	1029	375	38,225	0	38,225
87	SUCCESS	1029	3771	38,226	0	38,226
77	SUCCESS	1029	457	38,302	0	38,302
142	SUCCESS	1029	2064	38,535	0	38,535
128	SUCCESS	1029	3227	38,758	0	38,758

## Data Availability

The secondary and primary data used to support the findings of this study have not been made available because dataset confidentiality has to be maintained due to Ph.D. studies. However, both sample datasets have been incorporated into the article.
